# Adaptation of Temperate Seagrass to Arctic Light Relies on Seasonal Acclimatization of Carbon Capture and Metabolism

**DOI:** 10.3389/fpls.2021.745855

**Published:** 2021-12-02

**Authors:** Alexander Jueterbock, Bernardo Duarte, James Coyer, Jeanine L. Olsen, Martina Elisabeth Luise Kopp, Irina Smolina, Sophie Arnaud-Haond, Zi-Min Hu, Galice Hoarau

**Affiliations:** ^1^Algal and Microbial Biotechnology Division, Faculty of Biosciences and Aquaculture, Nord University, Bodø, Norway; ^2^Marine and Environmental Sciences Centre, Faculty of Sciences of the University of Lisbon, Lisbon, Portugal; ^3^Departamento de Biologia Vegetal da Faculdade de Ciências da Universidade de Lisboa, Lisbon, Portugal; ^4^Shoals Marine Laboratory, University of New Hampshire, Durham, NH, United States; ^5^Ecological Genetics-Genomics Group, Groningen Institute of Evolutionary Life Sciences, University of Groningen, Groningen, Netherlands; ^6^Marine Molecular Ecology Group, Faculty of Biosciences and Aquaculture, Nord University, Bodø, Norway; ^7^UMR MARBEC Marine Biodiversity Exploitation and Conservation, Univ Montpellier, CNRS, IFREMER, IRD, Montpellier, France; ^8^Ocean School, Yantai University, Yantai, China

**Keywords:** eelgrass (*Zostera marina*), climate change, Arctic light, respiration, photosynthesis, carbon capture, daylength, energy storage

## Abstract

Due to rising global surface temperatures, Arctic habitats are becoming thermally suitable for temperate species. Whether a temperate species can immigrate into an ice-free Arctic depends on its ability to tolerate extreme seasonal fluctuations in daylength. Thus, understanding adaptations to polar light conditions can improve the realism of models predicting poleward range expansions in response to climate change. Plant adaptations to polar light have rarely been studied and remain unknown in seagrasses. If these ecosystem engineers can migrate polewards, seagrasses will enrich biodiversity, and carbon capture potential in shallow coastal regions of the Arctic. Eelgrass (*Zostera marina*) is the most widely distributed seagrass in the northern hemisphere. As the only seagrass species growing as far north as 70°N, it is the most likely candidate to first immigrate into an ice-free Arctic. Here, we describe seasonal (and diurnal) changes in photosynthetic characteristics, and in genome-wide gene expression patterns under strong annual fluctuations of daylength. We compared PAM measurements and RNA-seq data between two populations at the longest and shortest day of the year: (1) a Mediterranean population exposed to moderate annual fluctuations of 10–14 h daylength and (2) an Arctic population exposed to high annual fluctuations of 0–24 h daylength. Most of the gene expression specificities of the Arctic population were found in functions of the organelles (chloroplast and mitochondrion). In winter, Arctic eelgrass conserves energy by repressing respiration and reducing photosynthetic energy fluxes. Although light-reactions, and genes involved in carbon capture and carbon storage were upregulated in summer, enzymes involved in CO_2_ fixation and chlorophyll-synthesis were upregulated in winter, suggesting that winter metabolism relies not only on stored energy resources but also on active use of dim light conditions. Eelgrass is unable to use excessive amounts of light during summer and demonstrates a significant reduction in photosynthetic performance under long daylengths, possibly to prevent photoinhibition constrains. Our study identified key mechanisms that allow eelgrass to survive under Arctic light conditions and paves the way for experimental research to predict whether and up to which latitude eelgrass can potentially migrate polewards in response to climate change.

## Introduction

Climate-change induced increases in surface temperatures are causing a worldwide poleward shift of temperature isotherms (McMahon and Hays, 2006) and concomitantly, distribution limits (Wernberg et al., 2013; Melbourne-Thomas et al., 2020), and spring phenology of species (Clausen and Clausen, 2013; Poloczanska et al., 2013). Furthermore, polar regions are warming at a particularly high rate (Høye et al., 2007; Burrows et al., 2011). For example, Arctic sea ice has melted at a rate of 6% per decade since the late 1970 (Cai et al., 2021), opening thermally suitable habitats with unoccupied niches available for colonizing species from the south (Cheung et al., 2009).

Whether a temperate species can immigrate into ice-free Arctic regions depends not only on finding thermally suitable conditions but also on its ability to tolerate extreme Arctic light conditions (Huffeldt, 2020). Constant daylight or darkness can be particularly challenging for sessile autotrophs. To what extent constant summer daylight inhibits cellular respiration (Kok effect; Griffin and Heskel, 2013; Heskel et al., 2013), growth, productivity, and photosynthesis in plants (Kallio and Valanne, 1975) remains controversial and may depend on the species (Sysoeva et al., 2010). A poleward range shift further implies that growth and reproduction are triggered in suboptimal seasons if temperate species follow the same photoperiodic changes as specific life cycle triggers when immigrating into the Arctic (Taulavuori et al., 2010). Yet, research and, thus, knowledge on physiological adjustments of temperate plants to Arctic light conditions is scarce in both terrestrial, and marine systems (Huffeldt, 2020).

Seagrasses comprise a polyphyletic group of marine flowering plants inhabiting shallow subtidal shores from subarctic to tropical regions. Seagrass meadows provide habitat and food that support a diverse food web, as well as important ecosystem services such as nutrient fixation, protection of the shoreline from erosion, nursery grounds for commercially important fishes, and carbon sequestration of ca. 104 Tg C year^−1^ (Duarte and Krause-Jensen, 2017), or 1% of the global annual carbon emissions (ca. 9.8 Gt C year^−1^; Friedlingstein et al., 2020). Thus, if seagrass can grow under Arctic light conditions, its predicted northward shift (Wilson and Lotze, 2019) will likely enrich biodiversity and introduce drastic functional changes in future Arctic coastal ecosystems, food webs, fish/invertebrate stocks, and biogeochemical cycles (Krause-Jensen and Duarte, 2014).

The eelgrass *Zostera marina* is the most widely distributed seagrass species, occurring in suitable habitats along shores of all five continents in the northern hemisphere (Green and Short, 2004). Eelgrass meadows grow in coastal waters from subtropical regions at latitudes as low as 36°N (e.g., southern Iberia; Green and Short, 2004) to polar regions at latitudes as high as *ca.* 70°N (e.g., Greenland at 64.5° N; Olesen et al., 2015) and northern Norway at 69.8° N (Olsen et al., 2013).

Temperature, optimally from 15–25°C (Boström et al., 2004; Staehr and Borum, 2011; Zhang et al., 2016), is considered as the most important factor controlling phases of eelgrass sexual reproduction (Blok et al., 2018). Photoperiod appears to not control eelgrass reproduction because the eelgrass genome lacks the phytochrome C gene (PHYC; Olsen et al., 2016) that is involved in the photoperiodic control of flowering in most terrestrial plants (Chen et al., 2014; Woods et al., 2014). Accordingly, the species’ northern distribution limit probably is limited by cool summer water temperatures <13.5°C that prevent seed maturation (Blok et al., 2018). As its seed germination is not regulated by photoperiodic rhythms (Moore et al., 1993), eelgrass seeds are likely to develop and disperse in a warming Arctic and contribute to a seed-driven northward shift of eelgrass meadows under projected climate change (Clausen et al., 2014; Blok et al., 2018). Temperature also is one of the most important factors for eelgrass growth (Clausen et al., 2014; Zhang et al., 2016). For example, increased investment in below-ground rhizomes contributes to larger annual biomass in populations from high latitudes compared with populations from warmer waters (Clausen et al., 2014).

Below-ground rhizomes provide stored energy to fuel eelgrass metabolism under low-light conditions during Arctic winter (Olesen and Sand-Jensen, 1993; Burke et al., 1996) and the concentration of stored carbohydrates in eelgrass beds increases with latitude (Colarusso, 2007; Novak et al., 2020). However, rising temperatures may lead to a mismatch between peak growth – advancing to winter darkness – and available solar radiation in spring (Clausen et al., 2014). The mismatch, in combination with an increased annual biomass turnover under warmer temperatures (Olesen et al., 2015), requires eelgrass to store sufficient resources to fuel winter metabolism and the higher and earlier production of shoots at the onset of the growing season (Clausen et al., 2014). Thus, poleward migrating eelgrass will have strong implications for the dynamics of below-ground carbon sinks.

The minimum light requirement for long-term survival and growth – meeting respiratory and metabolic energy demands – ranges between 11 and 34% surface irradiance (Ochieng et al., 2010), corresponding to 1.2–12.6mol photons m^−2^ day^−1^ (Gattuso et al., 2006). Six hours daily of saturating irradiance (Hk) was suggested to be the minimum allowing for eelgrass growth and survival (Dennison and Alberte, 1985). Furthermore, shaded eelgrass showed significantly higher mortality rates under increased temperatures (16% at 20°C vs. 0.57% at 12°C; Eriander, 2017).

Most previous research has focused on the impact of modest light reductions or depth limits of eelgrass (e.g., Ralph et al., 2007), although a period of 30 days at 10% surface irradiance (<3mol photons m^−2^ d^−1^) limited the survival of eelgrass transplants in estuarine temperate systems (Moore et al., 1997). The potential for poleward migration into the Arctic remains unknown because eelgrass survival under extreme light periods remains understudied. In order to investigate to what extent extreme Arctic photoperiods may present an energetic constraint to projected northward range shifts of eelgrass, we compared photosynthetic characteristics and gene expression patterns between two populations at the longest and shortest day of the year: (1) a Mediterranean population experiencing moderate annual fluctuations of 10–14 h daylength and 2) an Arctic population experiencing high annual fluctuations of 0–24 h daylength. The specific objectives were to:

1. Characterize energy-saving mechanisms (in photosynthetic and metabolic pathways) that may allow eelgrass to survive during long Arctic winter nights.
2. Determine possible adaptive mechanisms of Arctic eelgrass to compensate for energy loss during winter with increased investment in energy storing mechanisms during summer.

Differences in gene expression between summer and winter or day and night samples from a single population are interpreted as resulting from seasonal or diurnal acclimatization (Box 1). Our study cannot identify whether gene-expression or photosynthetic specificities of the Norwegian population (not replicated in the French population) are acclimatizations resulting from phenotypic plasticity or whether they are genetic adaptations (Box 1) that evolved under polar light regimes. Thus, in this manuscript, we use the term “adaptation” when referring to either acclimatization or genetic adaptation (Box 1).

BOX 1 **Glossary of adaptation-related terms**

**Acclimatization** – Phenotypic change over the life span of an organism induced by epigenetic regulation in response to environmental cues.

**Adaptation** – All plastic and genetically adaptive changes that reduce the negative effects of unfamiliar and undesirable external environment. This includes both acclimatization and genetic adaptation.

**Genetic adaptation** – Evolution of genetically determined heritable traits in a population across generations in response to natural selection.

## Materials and Methods

### Site Characteristics

Eelgrass shoots were sampled in Norway (Røvik, 67°16′06.2” N, 15°15′38.4″ E) and France (Sète, Thau Lagoon, 43°25′08.0” N, 3°40′03.9″ E). The youngest two shoots of each of six plants were collected at each site at noon and the following midnight around summer solstice (June 21) and winter solstice (December 21) 2017. Intact shoots were placed in RNAlater (ThermoFisher) and stored after 1–2 days at −20°C.

Defining Zeitgeber Times ZTs (time after an environmental time cue that entrains circadian rhythms, such as sunlight) that correspond between the French and Norwegian samples is complicated by the fact that the two latitudes do not only differ in diurnal light rhythms but also in light quantity and quality (Taulavuori et al., 2010) (i.e., light conditions at dawn at the French site do not correspond to dawn light conditions at the Norwegian site and, thus may mark different ZTs in the circadian cycle). We chose midday and midnight as time points to compare samples from the two locations as these represent times at which the plants from both locations had the potential to receive most/least light of the day in both seasons, and the time that is at equal temporal distance from sunset and sunrise. This implies, however, that differences between the Norwegian and French samples at corresponding time points may be partly explained by them being at different ZTs within their circadian rhythm. However, differences in ZTs are less affecting seasonal differences between the Norwegian and French, because we compared daily averages that involved measurements from both midday and midnight samplings.

Snapshots of temperature (°C) and light (Lux; Table 1) were taken at each sampling event in ca. 20 cm water depth with Pendant data loggers (Onset HOBO UA-002). Annual variation of temperature (°C), salinity, and Lux conditions at the Norwegian sampling site were recorded from spring 2016 to spring 2017 with data loggers (HOBO U24-002-C for salinity/temperature with 30 min intervals, and HOBO UA-002 for light/temperature with 10 min intervals) anchored ca. 30 cm above sandy substrate during low tide. At low tide, the light/temperature loggers were falling dry. Temperature (°C) and salinity conditions in the Thau Lagoon (43° 22′ 44.4” N, 3° 34′ 17.4″ E) were downloaded for year 2016 from the marine environmental station database of Thau Lagoon (Messiaen et al., 2018) on July 31, 2020, which were recorded every 30 min with data loggers (WTW) at 1–2m water depth. Light (PAR μmol m^2^ s^−1^) conditions were recorded with dataloggers (SKUW 215 PAR Sensor, Skye Instruments) every 30 min for December 2015–July 2016 at the Thau Lagoon Observatory (43°24′53” N, 3°41′16″ E; Trombetta et al., 2019) and were downloaded from Sea scientific open data publication (www.seanoe.org, accessed on July 31, 2020). To make the light conditions comparable between the Norwegian and French sampling sites, Lux values were converted to approximate PAR (photosynthetically active radiation) values by applying a multiplication factor of 0.019, an approximation for the spectral power distribution of sunlight (6,000 K; Creswell, 2010).

**Table 1:** Snapshots of temperature and light conditions measured at the sampling events.

Measurement	Population	June		December	
		Night	Day	Night	Day
Temperature (°C)	Norway (Røvik)	15.1	16.0	−1.9	−1.6
	France (Sète)	22.8	21.7	7.3	8.2
Light (μmolm^−2^ s^−1^)	Norway (Røvik)	6.3	235.6	0.0	0.0
	France (Sète)	0.0	327.2	0.2	535.4

**Figure 1:**
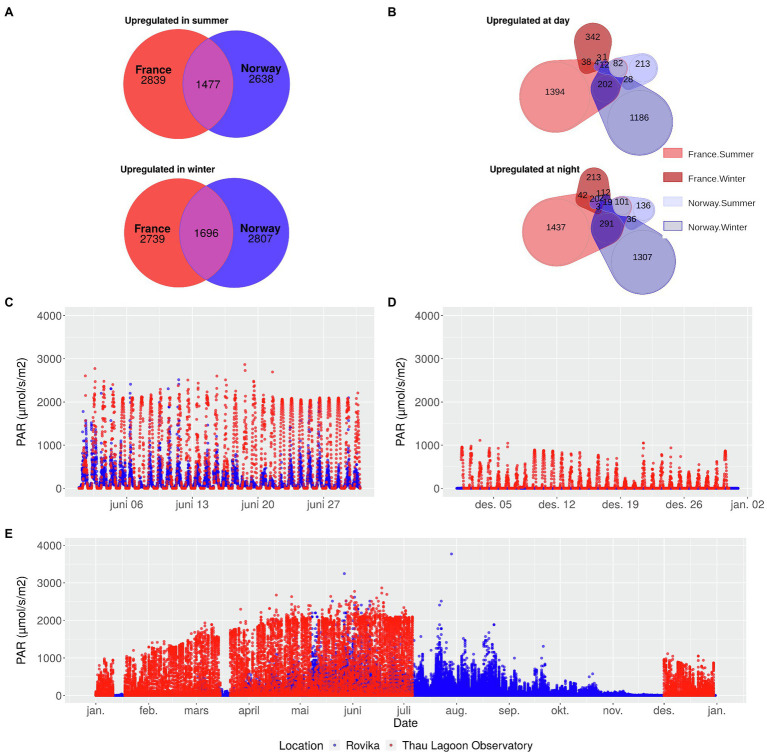
Seasonal and diurnal gene expression changes of the Norwegian and French population of the seagrass *Zostera marina* in relation to differences in annual light conditions at the sampling sites. Number of genes upregulated in summer, and winter **(A)** in France and in Norway, or upregulated during day, and night **(B)**, separately for summer and winter. The subset of genes in A and B are independent from each other but partly overlapping. Light levels in June **(C)**, December **(D)**, and over the entire year **(E)** at the Norwegian sampling site (blue) and at an observatory station in the Thau Lagoon close to the French sampling site (red). In the Thau Lagoon, values are missing from July to December. PAR: Photosynthetically active radiation.

Seasonal variation in light levels was greater in Norway, where PAR levels remained close to zero from mid-November to mid-January, but reached ca. 1,000 μmolm^−2^ s^−1^ in summer, similar to France (Figures 1C,D,E; Supplementary Table 1). In summer, light levels showed a diurnal rhythm in both Norway and France, but – as expected – reached slightly higher nocturnal levels in Norway.

Summer temperatures were ca. 10°C higher in France (20–25°C) compared to Norway (10–15°C; Supplementary Figure 1; Supplementary Table 1). Stronger Norwegian diurnal temperature variations likely reflected the differences between air and water temperatures around spring tides when the dataloggers may have been shortly exposed to air during low-tides but submerged during high-tides. In contrast, the eelgrass meadow in the Thau lagoon was constantly submerged. Accordingly, the data loggers revealed relatively constant salinity throughout the year at ca. 37 PSU (Supplementary Figure 2; Supplementary Table 6), while the high fluctuation (10–30 PSU) in Norway was due to seasonal rainfall, freshwater runoff, ice formation, and melting.

### Photosynthesis

At each sampling event, we assessed leaf photochemistry using a Chlorophyll *a* Pulse Amplitude Modulated (PAM) Fluorometer (FluorPen FP100, Photon Systems Instruments), by measuring the increase in chlorophyll *a* fluorescence upon illumination after a 10-min dark period induced by leaf-clips (OJIP curve; Bussotti et al., 2010; (Duarte et al., 2017). using a saturating pulse at 455 nm from 10 seagrass shoots.

When a dark-adapted leaf is illuminated with a saturating light intensity, it exhibits a so-called OJIP curve from a state where all reaction centers are opened (all quinone acceptors are oxidized and can accept electrons) to a state where all quinone acceptors are reduced and chlorophyll fluorescence reaches its maximum. The rise in fluorescence transient along this polyphasic OJIP curve depicts the rate of reduction kinetics of various components of Photosystem II (PSII). The OJIP curve provides information on the processes involved in photon absorption at the PSII antennae (ABS), transformation into an exciton and its free energy flux (RE), and transduction of its energy in the form of electronic potential vial electron transport (ET) from the donor side of the PSII to the electron acceptor of the PSI. The analysis of this OJIP transient (JIP test) allows to estimate the energy fluxes, involving absorption, entrapment in the PSII, transport to the quinone pool, and dissipation in the form of heat or fluorescence that helps to avoid photoinhibition damages. The formulae and definitions of terms derived from the JIP-test (chlorophyll *a* induction curves, or Kautsky curves measurements; Supplementary Table 2) are listed in Table 2.

**Table 2:** Formulae and definitions of terms derived from the JIP test.

Term	Formula	Definition
PI_ABS_	[*γ*_RC_^1^(1− *γ_RC_*)] × [*φ*_Po_^2^/(1− *φ*_Po_)] × [*ψ*_o_^3^/(1− *ψ*_o_)]	Performance Index reflecting the overall photosynthetic performance by combining three parameters: (1) the density of reaction centers, (2) the electron transport at the onset of illumination, and (3) the maximum energy flux reaching the reaction center in PS II.
ABS/CS	F_0_^4^	Absorbed energy flux measured as the minimal fluoresence intensity in a dark adapted frond when all reaction centers are opened (all quinone acceptors are oxidized and can accept electrons)
ET/CS	*φ*_Po_ × *ψ*_o_ × (ABS/CS)	Electron transport energy flux
TR_0_/CS	*φ*_Po_ × (ABS/CS)	Trapped energy flux
DI_0_/CS	(ABS/CS) − (TR_0_/CS)	Dissipated energy flux
Area	Extracted from the recorded OJIP	Size of the oxidized quinone pool; total complementary area between the fluorescence induction curve and F = F_M_
RC_0_/CS	*φ*_Po_ × (*V*_J_^5^/*M*_0_^6^) × ABS/CS	Density of photosystem II oxidized reaction centers per excited cross-section
TR_0_/DI_0_		Contribution of the light reactions for primary photochemistry
*Ψ*_0_/(1− *Ψ*_0_)		Contribution of the dark reactions for primary photochemistry

1 *γ*_RC_ = Chl_RC_/Chl_total_ = RC/(ABS + RC), the probability that a PSII Chl molecule functions as reaction center (RC).

2 *φ*_Po_ = TR_0_/ABS = [1−(*F*_0_/*F*_M_)], the maximum quantum yield for primary photochemistry, with *F*_M_ representing the maximum fluorescence intensity when all reaction centers are closed (all quinone acceptors are reduced).

3 *ψ*_0_ = ET_0_/TR_0_ = (1−V_J_), efficiency/probability for electron transport (ET), or the probability that an electron moves further than *Q*_A_ (the primary quinone acceptor).

4 *F*_0,_ minimal fluorescence when all PSII RCs are open.

5 *V*_J_ (*F*_j_−*F*_0_)/(*F*_M_−*F*_0_), with *F*_j_ representing the fluorescence intensity at the J-step (at 2ms).

6 *M*_0_ Slope of the origin of the fluorescence rise. Maximal rate of accumulation of the fraction of closed reaction centers.

Based on Zhu et al. (2005) and Duarte et al., (2017).

Differences in these photosynthetic characteristics between populations (day/night/season) were tested for significance with Wilcoxon rank sum tests followed by correcting the values of *p* for multiple pairwise comparisons using the Benjamini-Hochberg method with the R package “rstatix” v0.6.0 (Kassambara, 2020). Multivariate differences in photochemistry were estimated from photochemical raw data using a canonical analysis of principal coordinates (CAP) with Euclidean distances with the Primer 6 software (Clarke and Gorley, 2005). This multivariate approach is insensitive to heterogeneous data and frequently used to compare different sample groups (Duarte et al., 2017; Cruz de Carvalho et al., 2020). Differences in the overall photochemical metabolism were estimated from normalized photochemical raw data with a canonical analysis of principal coordinates (Duarte et al., 2017). Multivariate differences were visualized as Euclidean distances along the first two principal coordinates.

### RNA Preparation

RNA was extracted from small pieces of stored samples (RNAlater; ThermoFisher) that were homogenized (PowerLyzer 24; Qiagen) and processed according to the standard protocol of the RNeasy PowerPlant Kit (Qiagen). We consistently excised the mid portion of the youngest fully mature leaf in the shoot (usually the second-rank leaf), so that tissue-age dependent within- or among-leaf variations were unlikely to explain differences in gene expression (Ruocco et al., 2019a,b). RNA concentrations were measured with Qubit™ RNA HS Assays (ThermoFisher) using a Qubit 4 fluorometer. RNA integrity was verified on TapeStation 2200 with RNA ScreenTape. RIN values were >3.6.

RNA libraries were prepared from 1 μg of extracted RNA with NEBNext® Ultra™ II Directional RNA Library Prep Kit for Illumina (New England Biolabs) following the polyA mRNA workflow of the kit protocol. Libraries were verified on TapeStation 2200 using High Sensitivity D1000 ScreenTape. Paired-end (2 × 150 bp) RNA sequencing (RNASeq) data were generated using a NextSeq 500/550 High Output Kit v2 (Illumina) on an Illumina NextSeq 500 platform at Nord University.

### Sequence Data Analysis

Raw reads were trimmed for low quality (Phred score Q<20, 99% base call accuracy), and TruSeq adapter sequences (overlap > 3bp) before reads <25bp were removed with TrimGalore! v 0.6.0.^1^ Read quality was checked with FastQC v 0.11.8 to control for aberrant read base content, length distribution, duplication and over-representation.^2^

Trimmed reads were mapped to the genome of *Z. marina* (Olsen et al., 2016) v 2.2 from ORCAE (Sterck et al., 2012) and the organellar genomes (chloroplast and mitochondria) of *Z. marina* v2.1 from ORCAE (Sterck et al., 2012) with the splice-aware RNA-seq aligner STAR v 2.7.2a (sjdbOverhang 149; Dobin et al., 2013, 2016), guided by gff3 gene annotations v2.2 from ORCAE (Sterck et al., 2012), converted to gtf with the program gffread.^3^ Alignments that contained noncanonical splice junctions were filtered out. Duplication rates were checked with the R package “dupRadar” v1.14.0 (Sayols et al., 2016).

Read counts for 21,069 genes (Supplementary Table 3) were obtained with featureCounts in the R package “Rsubread” v1.34.6 (Liao et al., 2019) with the non-default settings minOverlap = 3, MinMQS = 20, strandSpecific = 2, isPairedEnd = TRUE. Genes of low expression (library average<5, *n* = 7,023) were excluded from subsequent analyses to avoid potential artefacts from sequencing errors.

Gene IDs of the *Z. marina* genome v2.2 (Olsen et al., 2016) were annotated with gene descriptions, Gene Ontology (GO) terms, KEGG Orthology groups (KO), and Enzyme Commission (EC) numbers (based on enzyme nomenclature) from the ORCAE database (Sterck et al., 2012). Gene ontology terms assign genes to one or more categories related to the molecular function, cellular component or biological process of the gene products. KEGG Orthology groups assign gene products to their function in a biological pathway of molecular interactions. The EC number classifies genes numerically based on the chemical reactions they catalyze.

### Multivariate Clustering of Gene Expression

To characterize overall transcriptomic differentiation, we clustered the samples hierarchically by the first five principal components of gene coverage (14,046 genes), which together explained 73.4% of the variation, using the R package “FactoMineR” v. 1.42 (Lê et al., 2008). For Principal Component Analysis (PCA), the scale.unit was set to FALSE not to scale the expression values to unit variance. Prior to PCA, we regularized log-transformed gene count values (Supplementary Table 4) with DESeq2 v 1.24.0 (Love et al., 2014) in order to assure homoscedasticity and to account for differences in sequencing depth.

### Differential Gene Expression

We tested for differential gene expression between summer and winter samples (day and night samples combined) and between day and night samples in France and Norway, respectively, with DESeq2 v1.24.0 (Love et al., 2014). The values of *p* were adjusted with Independent Hypothesis Filtering based on the R package “IHW” v1.14.0 (Ignatiadis et al., 2016). We plotted overlaps of differentially expressed genes between seasons and locations with the R package “eulerr” v6.1.0 (Larsson, 2019) and “nVennR” v0.2.1 (Pérez-Silva et al., 2018).

For each population, we tested (Fisher’s exact test) for enriched gene ontology terms (biological process, cellular component, and molecular function) in genes that were differentially expressed between day and night samples or between summer and winter samples with the R package “topgo” (Alexa and Rahnenführer, 2010). Significance levels were adjusted with the Benjamini and Hochberg correction (Benjamini and Hochberg, 1995), using the p.adjust function in R (R Core Team, 2019), to control for the false discovery rate when conducting multiple comparisons.

The adjusted-log10 of the values of *p* of significantly enriched GO-terms were visualized for each population in heatmaps using the heatmap.2 function of the R package “gplots” v.3.0.3 (Warnes et al., 2020). GO terms were hierarchically clustered with the R package “goSTAG” v1.12.0 (Bennett and Bushel, 2017) based on euclidean distances and Ward’s minimum variance clustering method with Ward’s (Ward, 1963) clustering criterion implemented (ward.D2 option; Murtagh and Legendre, 2014). The sample dendrogram was based on Euclidean distances and a complete linkage clustering method.

Enriched/perturbed KEGG metabolic pathways in the fold-change matrices of differentially expressed genes were identified using the R package “gage” v2.37.0 (Luo et al., 2009). Nuclear genes were mapped to KO (Kegg Orthology)-terms by translating EC numbers from the genome annotation to corresponding KO-terms based on the KEGG API list http://rest.kegg.jp/link/ec/ko. For organellar genes, EC numbers and, thus KO-terms were not annotated. For pathways perturbed (*p* < 0.05) in at least one of the populations and seasons, we plotted the significant fold-changes of molecular functions (KO-terms) for all populations and seasons with the R package “pathview” v3.11 (Luo and Brouwer, 2013). When several genes referred to the same KO-term, we plotted their average fold-change in the respective pathway.

## Results

### Photosynthesis

**Figure 2:**
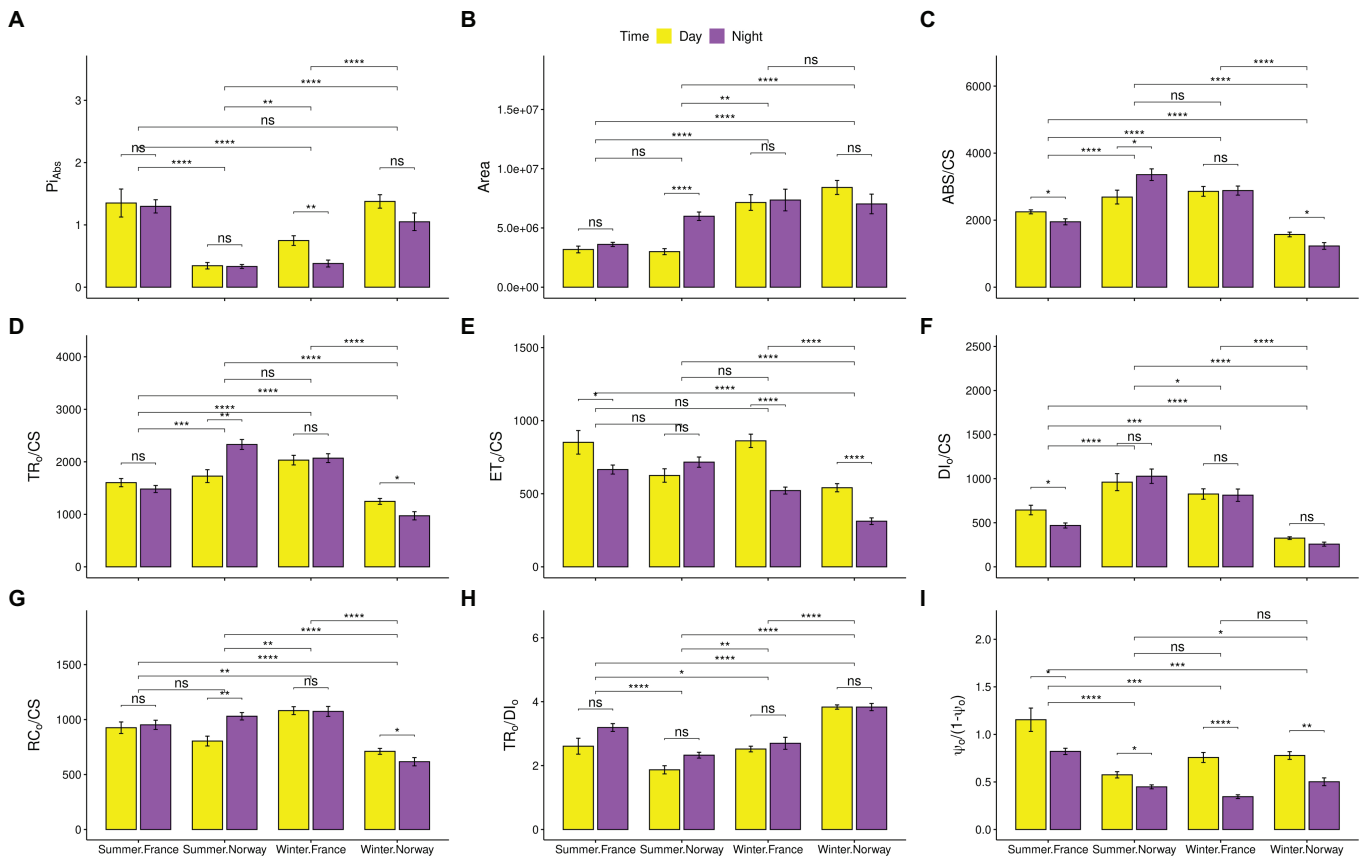
Photosynthetic characteristics. Snapshots of photosynthetic characteristics with standard error bars and indications for significant differences between populations, day and night, or summer and winter. Significance codes after the value of *p* correction (Benjamini-Hochberg) for multiple comparisons: “<0.05”: *, “<0.01”: **, “<0.001”: ***, “<0.0001”: ****. **(A)** Index of photosynthetic performance (PI_ABS_). **(B)** Size of the oxidized quinone pool (Area). **(C)** Absorption per excited cross-section (ABS/CS). **(D)** Trapping per excited cross-section (TR_0_/CS). **(E)** Electron transport per excited cross-section (ET_0_/CS), **(F)** Dissipated energy flux per excited cross section (DI_0_/CS). **(G)** Oxidized reaction centers per excited cross-section (RC_0_/CS). **(H)** Contribution of the light reactions to the primary photochemistry (TR_0_/DI_0_). **(I)** Contribution of the dark reactions to the primary photochemistry [Ψ_0_/(1-Ψ_0_)].

Overall photosynthetic performance, represented by the photochemical performance index (PI_ABS_; Figure 2A) showed inverse seasonal shifts in Norway and France. While the French population performed significantly better during summer, the Norwegian population performed significantly better during winter. The reduced summer performance of the Norwegian population was associated with peculiar photosynthetic characteristics. In contrast to the French population and the Norwegian winter samples, light harvesting ability increased in the Norwegian summer samples under non-saturating light levels during night-time, as represented by the increase in absorbed (ABS/CS; Figure 2C) and trapped (TR_0_/CS; Figure 2D) energy flux, and in the number of oxidized reaction centers per leaf cross section (RC_0_/CS; Figure 2G). The contribution of dark reactions to the overall photochemistry [Ψ_0_/(1-Ψ_0_); Figure 2I] decreased significantly during summer nights. However, despite readiness of the electron transport chain (ETC) for electron transport, as indicated by the significant increase in the size of the oxidized quinone pool during Norwegian summer nights (Area; Figure 2B), the electron transport energy flux (ET_0_/CS; Figure 2E) did not increase significantly. During winter, despite a significant increase in the contribution of light and dark reactions to the primary photochemistry [TR_0_/DI_0_, and Ψ_0_/(1−Ψ_0_); Figures 2H,I], the Norwegian population showed a significant reduction in all energy fluxes, particularly during night-time (ABS/CS, TR_0_/CS, and ET_0_/CS; Figures 2C–E), including dissipated energy loss (DI_0_/CS; Figure 2F).

**Figure 3:**
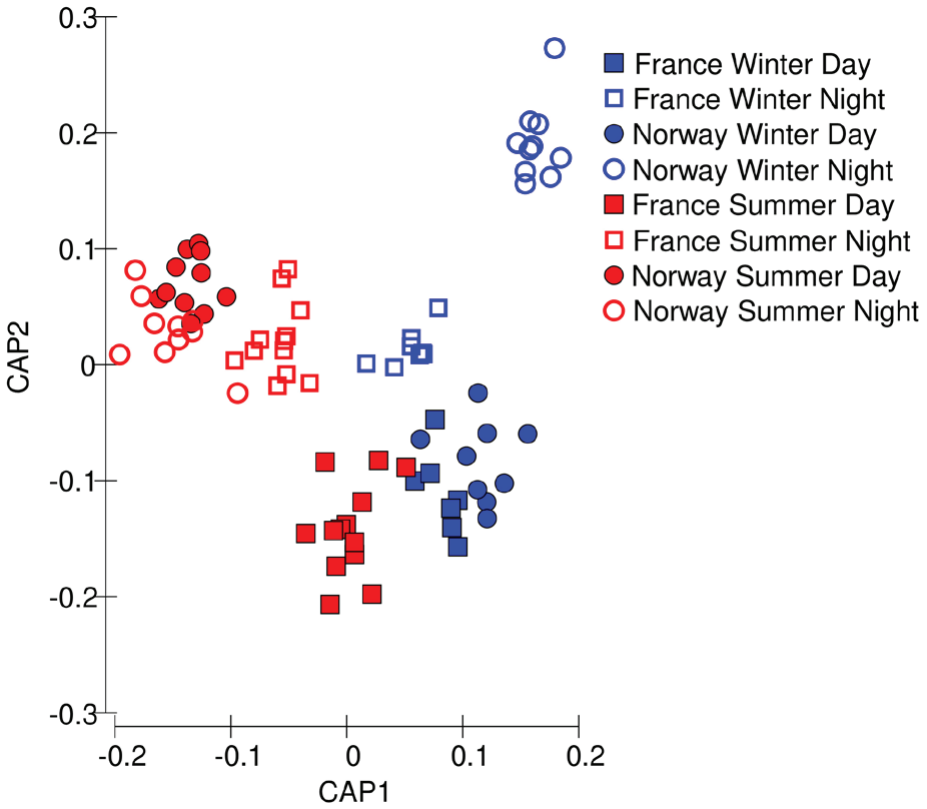
Multivariate differences among samples in photochemical data, visualized as Euclidean distances along the first two principal coordinates.

The samples clustered well by sampling location and daylength (78 of 80 samples correctly identified) along the first two principal components showing that the photochemical traits from the chlorophyll *a* induction curves could well classify the samples (Figure 3). The Norwegian summer samples grouped into one cluster, irrespective of whether they were collected at day or night. The Norwegian winter samples collected during nighttime formed another distinct cluster. Winter daytime samples from France and Norway grouped close together, indicating that winter had a prevalent effect over location.

### Gene Expression

**Figure 4:**
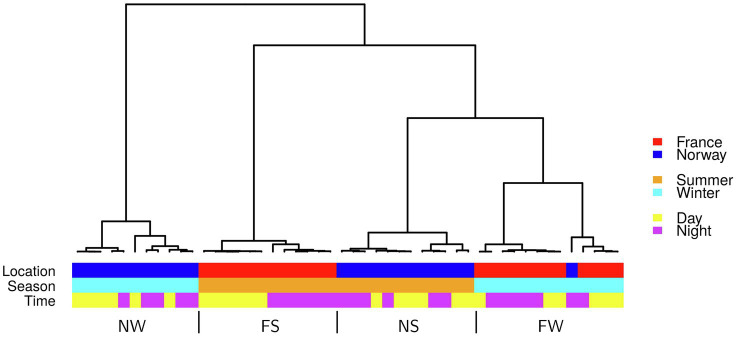
Hierarchical cluster of all 48 samples based on the first five principal components of expression in 13,932 genes. FS: France Summer, FW: France Winter, NS: Norway Summer, NW: Norway Winter.

On average, 76% of 1 million raw reads (average per library) mapped to exons (Supplementary Table 5). Based on the expression of 13,932 genes covered >5 times, the samples grouped into four clusters, separated by location (France/Norway) and season (Summer/Winter; Figure 4). The most distinct gene expression patterns were recorded in the winter samples from Norway (NW). Gene expression was most similar between winter samples from France (FS) and summer samples from Norway (NS; Figure 4). Strictly distinct clusters of gene expression between day and night were only observed in France during summer (FS).

Seasonal differences in gene expression were found in 8652 genes in the French population and in 8569 genes in the Norwegian population (Supplementary Table 6). In summer, 4,274 genes were upregulated in France, and 4,084 genes in Norway, of which 1,475 overlapped between the two populations (Figure 1A). In winter, 2,691 genes were upregulated in France, and 2,798 genes in Norway, of which 1,687 overlapped between the two populations (Figure 1A).

Diurnal differences in expression were strongest (>3,000 genes) in, and most similar (>500 genes shared) between the French summer samples and the Norwegian winter samples (Figure 1B). In contrast, the number of genes with diurnal expression differences was an order of magnitude lower (ca. 600 genes) in the French winter samples or the Norwegian summer samples (Figure 1B). All genes with diurnal differences in gene expression are characterized in Supplementary Table 7.

### Functional Changes

**Figure 5:**
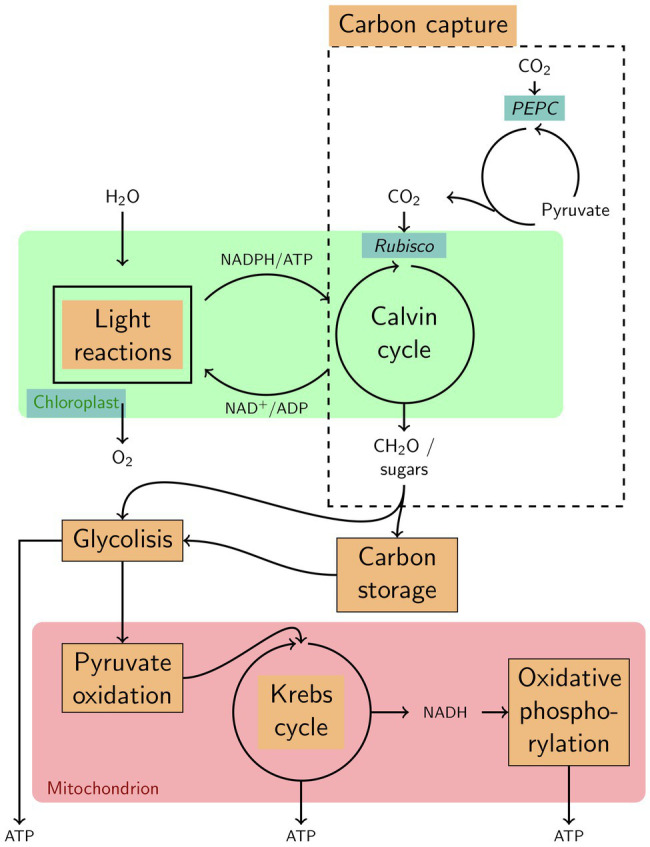
Highly simplified overview of various functions that characterize the specificities in seasonal variation of gene expression in the Norwegian population of *Zostera marina*. Functions, enzymes and components upregulated in winter are highlighted with a blue background; such upregulated in summer are highlighted with an orange background.

Genes involved in photosynthesis, carbon fixation (dark reactions of photosynthesis; Supplementary Figure 3), and sugar metabolism (fructose and mannose metabolism: Supplementary Figure 4 and glycolysis: Supplementary Figure 5) showed contrasting seasonal patterns between the Norwegian and French seagrass populations. Those are likely to explain the functional basis of the strong transcriptome similarity between Norwegian winter samples and French summer samples (Figure 4). Summer upregulation/winter downregulation of most of the organellar functions, both related to the chloroplast and the mitochondrion, characterizes the functional transcriptome specificities of the Norwegian samples (Figure 5). All pathways enriched for seasonal or diurnal differentially expressed genes are listed in Supplementary Table 8.

#### Photosynthesis and Carbon Sequestration

**Figure 6:**
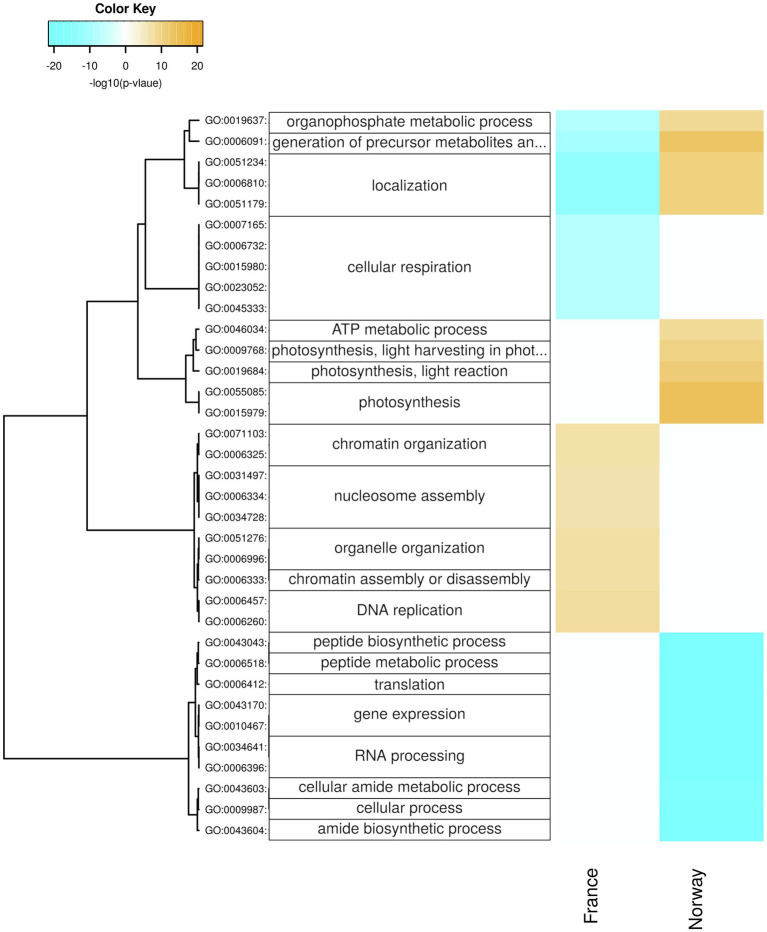
Seasonal changes in biological processes. Enriched processes are shown for each population with positive-log10 (*p*) for genes upregulated in summer (orange) and with negative-log10 (*p*) for genes upregulated in winter (cyan). The hierarchical cluster of GO terms is based on Euclidean distance and a ward.D2 clustering algorithm (Ward’s minimum variance method).

**Figure 7:**
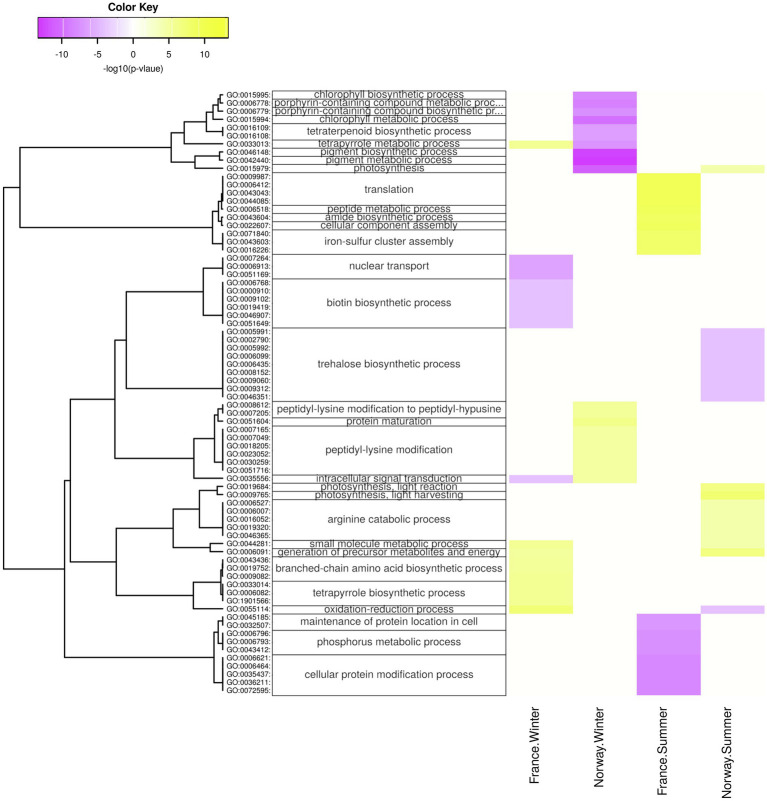
Diurnal changes in biological processes. Enriched processes are shown for each population x season with positive-log10 (*p*) for genes upregulated at day (yellow) and with negative-log10 (*p*) for genes upregulated at night (violet). The hierarchical cluster of GO terms is based on Euclidean distance and a ward.D2 clustering algorithm (Ward’s minimum variance method).

The Norwegian samples upregulated in summer genes related to photosynthesis and ATP metabolism (Figure 6; Supplementary Figure 6), photosystem, and photosynthetic membrane (Supplementary Figure 6), especially during the day (Figure 7; Supplementary Figures 7,8). Moreover, the Norwegian summer samples showed enrichment (*p* < 0.05) of genes with significant upregulation in the fructose and mannose metabolism (ko00051; Supplementary Figure 4). While significantly lower expressed in winter, genes involved in photosynthesis (Figure 7), chlorophyll binding (Supplementary Figure 7), chloroplast, photosystem, and photosynthetic membrane (Supplementary Figure 8) were significantly upregulated in winter nights as compared with winter days. During summer nights, the Norwegian samples showed the upregulation of trehalose biosynthesis (Figure 7).

The carbon fixation pathway in photosynthetic organisms (ko00710; Supplementary Figure 3) was enriched (*p* < 0.05) for genes that were upregulated in the Norwegian samples during summer but in the French samples during winter. The French samples upregulated during winter days genes involved in oxidoreduction (Figure 7; Supplementary Figure 7), as well as chloroplast genes (Supplementary Figure 8), and were enriched for upregulated genes in the pentose phosphate pathway (ko00030; Supplementary Figure 9).

#### Respiration

The French samples upregulated in winter genes related to respiration, including GO-terms of cellular respiration (Figure 6), mitochondria (Supplementary Figure 6), and oxidoreductase activity (Supplementary Figure 10). Moreover, the French winter samples were enriched for genes involved in glycolysis/gluconeogenesis (ko00010; Supplementary Figure 5).

In contrast, the Norwegian upregulated genes related to mitochondria (Supplementary Figure 6) and showed enrichment (*p* < 0.05) of genes with significant upregulation in glycolysis/gluconeogenesis (ko00010; Supplementary Figure 5) in summer. Only genes involved in the mitochondrial inner membrane were upregulated in the Norwegian samples during winter (Supplementary Figure 8).

#### Peptide Synthesis

In winter, the Norwegian samples upregulated functions and processes involved in gene expression, including peptide synthesis, translation, RNA processing (Figure 6), binding of nucleic acids and catalytic activities on RNA and tRNA (Supplementary Figure 10), as well as genes involved in intracellular organelles such as the ribosome (Supplementary Figure 6). During winter days, the Norwegian samples had upregulated genes involved in protein maturation, peptidyl-lysine modification (Figure 7), NAD^+^ kinase, and phosphatidyl-inositol phospholipase C activity (Supplementary Figure 7).

The French samples showed diurnal changes in the expression of genes related to protein synthesis during summer, when genes involved in translation, amid biosynthesis, (Figure 7), as well as the ribosome (Supplementary Figure 8) were upregulated during the day.

## Discussion

Our results provide the first assessment of seasonal (and diurnal) changes in photosynthetic and genome-wide gene expression patterns specific to a population growing above the Arctic circle that contrast patterns observed in a Mediterranean (control) population. Our results indicate that adaptation to polar light regimes involves continued photosynthesis, carbon fixation and energy conservation during long winter nights, and carbon storage during the polar summer.

### Strategies to Survive Long Winter Nights

#### Photosynthesis and Carbon Fixation in Winter

Despite reduced rhythmicity in daylight levels during the Arctic winter, our Norwegian winter samples appeared to still follow a day-night rhythm in gene expression (Figure 1B). Indeed, the Norwegian population showed, in contrast to the French population, a far higher number of genes with diurnal expression differences during winter (3,099; Figure 1B) than during summer (657; Figure 1B). Increased night-time expression of genes related to the synthesis of chloroplasts and chlorophyll (Figure 7) is in line with earlier field observations of enhanced chlorophyll in winter acclimatized eelgrass (Thom et al., 2008), and suggests that the Norwegian population actively synthesizes photosynthesis-relevant proteins to capture the smallest amounts of photosynthetically active radiation in winter.

Efficient use of very dim Arctic winter light in the Norwegian samples is suggested by a significant increase in the photochemical performance index (PI_ABS_; Figure 2A), and in the contribution of both light and dark reactions (Figures 2H,I) to the primary photochemistry during winter. Adjustments that may contribute to photosynthesis under low light levels include an increase in chlorophyll *b* levels in comparison to chlorophyll *a*, thus increasing photosystem II antennae size, and maximizing the photon harvesting probability (Ort et al., 2011) with a simultaneous increase (by chlorophyll *b*) in the affinity for harvesting light of long wavelengths that protrudes to greater water depths (Lee et al., 2007; Eriander, 2017). Moreover, under low light levels, eelgrass plants invest more energy into vertical leaf growth, compensating the low light levels with large photosynthetic surfaces and a reduction in canopy depth (Ochieng et al., 2010; Wong et al., 2021). While energy fluxes (ABS/CS and TR_0_/CS; Figures 2C,D) during the Norwegian winter cannot match fluxes occurring in summer, they are likely to prevent the depletion of assembled carbohydrate storages. Accordingly, winter survival of eelgrass at its lower depth distribution in the Great Bay Estuary (New Hampshire, United States), depended primarily on adaptations that allowed for continued photosynthesis under extreme low light conditions (e.g., increase in leaf size, chlorophyll a, and maximum quantum yield of PSII; Chapter IV in Ochieng, 2008). Thus, adaptation to the Arctic appears to involve seasonal acclimatization that allows to actively use the smallest amounts of winter light *via* photosynthesis.

Accordingly, the Norwegian population appears to present the physiological potential to capture carbon in winter. For example, ribulose-bisphosphate carboxylase (RuBisCO, K01601), the initial carboxylase of the Calvin cycle that fixes atmospheric CO_2_, was expressed at higher levels during winter than during summer (Figure 5; Supplementary Figure 3). This contrasts with 30% under-expression of RuBisCO in *Posidonia oceanica* leaves adapted to low light-levels (Mazzuca et al., 2009) and shows that winter gene expression in an Arctic population is not comparable with gene expression characteristics of seagrass experiencing low light levels in turbid waters year-round. In addition, the enzyme RuBisCO activase, although showing significantly higher expression in the Norwegian population during summer (Zosma13g01130, Supplementary Table 6), was still expressed during winter (Zosma294g00110, Zosma13g01130, and Zosma88g00030 in Supplementary Table 7). The presence of this activator, which removes the RuBisCO inhibitor CAP (2-carboxylarabinitol-1,5-bisphosphate), suggests that the Norwegian population could indeed maintain carbon fixation during the dark winter period. If increased RuBisCO expression in Norwegian winter samples is related to cold-acclimation, as shown in maize (Salesse-Smith et al., 2020), then warmer winters could hinder carbon assimilation and, thus, survival in the future Arctic.

Furthermore, the Norwegian winter samples had upregulated the enzyme phosphoenolpyruvate carboxylase (PEPC, K01595; Figure 5), which can catalyze CO_2_ fixation with phosphoenolpyruvate to produce oxaloacetate and inorganic phosphate. This suggests that *Z. marina*, although being characterized as a C3 plant that fixes CO_2_ primarily *via* RuBisCo (Koch et al., 2013), can fix some carbon during winter independent from photosynthesis, although at much lower levels than RuBisCo would allow (Raven and Farquhar, 1990). PEPC CO_2_ fixation is not uncommon in C3 plants (Chi et al., 2014), given that enzymes involved in the C4 photosynthetic biochemistry have functions in C3 plants other than in C4 photosynthesis, including the supply of carbon skeletons (*via* the intermediates oxaloacetate and malate) to the citric acid cycle (Lepiniec et al., 1994; Aubry et al., 2011; Figure 5) for the formation of new tissue. Thus, we suggest that PEP-carboxylase may present a key enzyme that allows for winter growth by decoupling photosynthesis and biomass formation not only in polar algae (Wiencke et al., 2007, 2009) but also in Arctic eelgrass.

Active growth in winter would explain the presence of freshly grown seagrass leaves in the dark season in the Norwegian population (Supplementary Figure 11). Eelgrass reaches light saturation between ca. 70 and 900 μmolm^−2^ s^−1^, and compensation above ca. 1–36 μmolm−^2^s^−1^ (Ochieng, 2008). Given that photosynthetically active radiation exceeded 10 μmolm^−2^ s^−1^ in the Norwegian population only for short mid-day periods on two December days in 2016 (Supplementary Table 1), photosynthesis-driven carbon dioxide assimilation is expected to be very low in the Norwegian winter.

Winter growth, therefore, is expected as a result of light-independent carbon fixation and the usage of stored carbohydrates mobilized from below-ground tissues (Burke et al., 1996). For example, strong activation of sucrose synthase, of which the products can drive many metabolic pathways, has been shown in sink-tissues of eelgrass under negative carbon balances (Alcoverro et al., 1999). Sustained growth during periods of negative carbon balance (respiratory C loss exceeding photosynthetic C gain) – fueled by mobilized carbohydrate storages – has been shown in both eelgrass (Wium-Andersen and Borum, 1984; Olesen and Sand-Jensen, 1993), and the Mediterranean seagrass *P. oceanica* (Alcoverro et al., 2001). In conclusion, our results suggest that the use of low light levels and light-independent carbon fixation count among the adaptations that allow for eelgrass to grow during winter without fully depleting their stored carbohydrates.

#### Energy Conservation Strategies in Winter

The Norwegian population appears to conserve energy during winter, as indicated by a significant reduction in all photosynthetic energy fluxes (ABS/CS, TR_0_/CS and ET_0_/CS; Figures 2C–E), resulting in a significantly reduced loss of energy *via* dissipation (DI_0_/CS; Figure 2F). Such state of metabolic dormancy, limiting the enzymatic reactions of photosynthesis, may be adaptive to the combination of low light availability and low winter temperatures.

Moreover, the respiratory breakdown of carbohydrates must have been reduced in the Norwegian population during winter, given that genes related to glycolysis, ATP metabolism (Figure 6; Supplementary Figure 5), and the fructose and mannose metabolism (Supplementary Figure 4; e.g., fructose-bisphosphate-aldolase K01623; Figures 6, 7) were downregulated. Accordingly, while upregulated during winter in the French population (Figure 6), genes involved in cellular respiration were significantly downregulated in the Norwegian population during winter (e.g., malate dehydrogenase, pyruvate dehydrogenase, and Cytochrome c oxidase subunit 5b; Supplementary Table 6; Figure 6), suggesting a shutdown of catabolic pathways during the Norwegian winter. Thus, our results provide the first molecular support for winter-acclimatization in eelgrass that explains an increase in photosynthesis-to-respiration ratios, in line with reports of a greater net primary productivity in eelgrass acclimatized to winter than to summer conditions (Thom et al., 2008). Acclimatization of eelgrass to low light conditions, further involves reduced rhizome and root growth, lower production of new shoots through lateral branching, shoot density, biomass, and sheath length (Ochieng et al., 2010; Wong et al., 2021), all of which reduce the respiratory burden. Furthermore, active reduction in respiration rates likely accompanies other factors, such as increased chlorophyll *a* content and lower epiphytic growth, in photo-adaptive processes that are involved in lowering light-saturation and compensation points in winter-acclimatized eelgrass (Ochieng, 2008; Thom et al., 2008). Such seasonal acclimatization may to some degree counteract the respiratory burden of rising temperatures by decreasing respiration rates and critical light requirements (Zimmerman et al., 1989; Staehr and Borum, 2011).

Glycolysis (Figure 5; Supplementary Figure 5) is a catabolic pathway providing energy in the form of NADH, ATP, and pyruvate, the latter being further catabolized in the citric acid cycle as part of oxidative respiration. Its downregulation during winter in this study contrasts with upregulation of genes involved in carbohydrate catabolism (e.g., 1-fructose-bisphosphate aldolase) in low-light adapted seagrass leaves of the species *P. oceanica* (Mazzuca et al., 2009). Downregulation in our study may be explained by the ability of our low-light (winter) eelgrass to build up carbon storages during summer and, thus, to obtain carbon-storages during winter from storage tissues such as roots and rhizomes, instead of from the above-ground leaf tissue. In other words, while carbon catabolism was low in the seagrass leaves, we cannot know what processes were active in the below-ground organs, in which sucrose and starch can be depleted by 50 and 23%, respectively, after a 3-week shading period (Eriander, 2017).

That carbon storages were built up over summer was suggested by the significantly increased expression of enzymes involved in carbon fixation (Supplementary Figure 3) and by significantly increased summer expression of two genes encoding for sucrose-phosphate synthase (Zosma255g00080, Zosma87g00120, Supplementary Table 6). This enzyme is involved in the synthesis of sucrose, the dominant storage carbohydrate in seagrasses (Touchette and Burkholder, 2000). Reduced carbohydrate cleavage in the sampled winter leaves is likely to contribute to the ability of the Norwegian population to survive long dark periods as the reduced demand for metabolic energy prevents depletion of carbon storages.

### Carbon Storage and Stress Under Polar Summer Light

Upregulation of photosynthesis-related genes in the Norwegian population during summer (Figure 6) was also shown in the seagrass *P. oceanica* when exposed to high light levels (Dattolo et al., 2014) and is likely to enable the plants to process the increased amount of captured light energy (Walters, 2005). Upregulation of genes with functions in carbon capture during summer in the Norwegian (but not the French) samples (Supplementary Figure 3) is necessary for the rapid build-up of stored carbohydrates during the short summer period, and provides a molecular explanation for increasing autumn starch content with latitude (in *Zostera noltii*; Soissons et al., 2018).

Surprisingly, the light intensity at the Norwegian sampling site showed a clear day-night rhythm during summer. Even though the summer sun does not set below the horizon, shading by mountain ridges and the low sun angle during night-time appears to create a short, but clearly noticeable drop in available photosynthetically active radiation. This diurnal light rhythm was paralleled by diurnal changes in gene expression. For example, the daytime upregulation of arginine catabolism (Figure 7) may mobilize nitrogen storages and thus, allow for active growth under nitrogen limitation (Winter et al., 2015). In addition, the nighttime upregulation of trehalose biosynthesis (Figure 7), in which the intermediate compound trehalose-6-phosphate acts as a sensor for available sucrose, is likely involved in the regulation of sucrose-levels that have built up over the day (Lunn et al., 2014; Figueroa and Lunn, 2016) and plays a role in plant growth, development, and protection against various environmental stressors (Lunn et al., 2014; Joshi et al., 2020). Thus, the diurnal changes in gene expression during summer are likely related to active growth and processing of assembled sugars. Local geography, e.g., the presence of mountain ridges, may play an important role in allowing eelgrass to process a dark phase in high latitudes.

Despite diurnal fluctuations in daylight levels, the lack of complete darkness during summer significantly lowers the photosynthetic performance (PI_ABS_) of the Norwegian samples (Figure 2A), in contrast to the seasonal change in photosynthetic performance in the French population (with significantly higher values during summer: Figure 2A). Particularly during summer nights, the Norwegian population showed a significant increase in absorbed (ABS/CS; Figure 2C), trapped (TR_0_/CS; Figure 2D), and electron transport (ET_0_/CS) energy fluxes, as well as the size of the oxidized quinone pool (ABS) and the number of oxidized reaction centers per leaf cross section (RC_0_/CS; Figure 2G). Although these photosynthetic adjustments increase the light harvesting ability and efficiency to use low-light, the Norwegian samples also showed a significant increase in energy dissipation (DI/CS), suggesting an inability to use excessive amounts of light energy in summer.

Low light intensities during the polar summer nights appear to have allowed the Norwegian plants to process a dark phase to recycle intermediates of the Krebs cycle (such as malic acid) that are consumed during the light phase of photosynthesis. The dark phase is triggered by an inhibitor (2-carboxylarabinitol-1,5-bisphosphate, CAP) that is only produced under low light when the chloroplast pH shifts from acid to basic. It was not found in any of the genes showing diurnal differential expression (Supplementary Table 7). A crucial limit to the northward shift of seagrass may be set by the latitude at which the sunlight is too strong to allow the procession of a dark phase. The existence and identification of this latitude, however, relies on future studies involving transplant experiments to a range of increasing latitudes.

### The Metabolic Dilemma of Carbon Storage Tissue

Our findings suggest that eelgrass can store sufficient energy reserves to survive dark periods lasting longer than those at 67°N (our Norwegian sampling site). In fact, if sugar levels were depleted in mid-winter, we would have expected to see an upregulation of genes involved in protein degradation, as autophagic protein degradation has been suggested as an alternative energy source under sugar depletion in eelgrass (Mochida et al., 2019). As the expected upregulation was not observed, a poleward shift of eelgrass should not be hindered by the potential to store sufficient photosynthates. In order to test this hypothesis, future studies must evaluate carbohydrate reserves in autumn, a good indicator of winter survival in the seagrass *Zostera noltii* (Govers et al., 2015), and reserve depletion over long dark periods directly in below-ground rhizomes.

While energy storing rhizomes allow eelgrass to survive dark periods, they also represent respiratory burdens that can increase light requirements, potentially preventing poleward migration (Olesen and Sand-Jensen, 1993; Lee et al., 2007). This is particularly true as respiration increases under rising temperatures (Lee et al., 2007; McMinn and Martin, 2013), faster than photosynthesis (higher Q10; Thom et al., 2008; Staehr and Borum, 2011). The respiratory demands of seagrass roots and rhizomes explain the relatively high amounts of light (10–30% surface irradiance; Ochieng et al., 2010) required for long-term survival, as compared with 1–5% for many species of phytoplankton and algae (Kenworthy and Fonseca, 1996). Below-ground rhizomes in eelgrass consume up to 15% of the carbon fixed, and up to 25% at the deepest range-margin (Kraemer and Alberte, 1993). Moreover, even if carbohydrate concentrations are not depleted, seagrass originating from 36°N cannot survive extreme experimental winter light limitation (2 h irradiance saturated photosynthesis), presumably through the effects of anoxia on increased translocation of energy reserves (Alcoverro et al., 1999). Thus, increasing carbohydrate storage does not guarantee long-term survival in the dark.

The need of eelgrass to elevate carbon storage in warming Arctic oceans may be lowered by thermal acclimatization that increases respiratory energy efficiency, as has been shown in terrestrial Arctic tundra species (Kornfeld et al., 2013), or by increasing CO_2_ levels (Zimmerman et al., 1997). Eelgrass is generally carbon limited, because it cannot efficiently use the inorganic carbon from the water column (HCO3-; Lee et al., 2007). Thus, increasing CO_2_ levels are expected to increase photosynthetic rates without affecting respiration (Zimmerman et al., 1997). If turbidity does not hinder long-term survival (Moore et al., 1997) in the future Arctic, increasing CO_2_ levels will reduce the need for producing high below-ground biomass by shortening the seasonal period during which eelgrass experiences a negative carbon balance.

### Conclusion and Perspectives

In this study, we show that distinct seasonal expression patterns of an eelgrass population from the Norwegian subarctic are driven by genes encoding functions of the organelles (chloroplast and mitochondrion). These are likely to provide key physiological adaptations that allow eelgrass to endure the polar dark season, and suggest metabolic strategies to enhance restoration success in turbid waters. The two most prominent features were:

1. Active carbon capture in winter. Winter expression of genes encoding for functions related to carbon capture, including Rubisco and PEP-carboxylase (Figure 5), may allow for eelgrass growth and carbon fixation during the polar dark season. This resembles the season-anticipator strategy of polar algae to fix carbon and grow even under the ice during Arctic darkness when nutrient conditions are highest (Berge et al., 2015).
2. Metabolic dormancy in winter. Downregulation of genes related to cellular respiration/carbohydrate cleavage provides the first molecular support for winter-acclimatization in eelgrass that explains an increase in photosynthesis-to-respiration ratios as compared with summer conditions (Thom et al., 2008) and is likely to prevent depletion of carbohydrate storages during the dark period.

This study alone, based on two meadows and an assessment of metabolic and transcriptomic responses, does not allow us to predict whether eelgrass will be able to migrate poleward into a warming, ice-free Arctic. Although this study reveals specificities that are functionally relevant at the species’ current northernmost range of distribution, its potential to migrate further poleward depends on the plastic and/or adaptive potential of the identified key functions. While genetic adaptations would rely on standing genetic diversity that allows for evolutionary change over several generations, acclimatization – which can build up within the lifetime of an organism (Kelly et al., 2012; Donelson et al., 2019) – would allow eelgrass to immediately occupy niches that open up in the Arctic. For example, in the seagrass *Thalassia testudinum*, phenotypic variation among subpopulations across an environmental gradient in the Florida Bay were mainly ascribed to phenotypic plasticity because gene flow was high within 50 km distance (Bricker et al., 2011). Further studies are needed to identify to what degree the north–south differences that we identified result from acclimatization or genetic adaptation (Box 1):

1. Reciprocal transplantation of individuals between northern to southern latitudes would allow to identify genetic adaptations as features in photosynthesis and gene expression as such remaining distinct in northern/southern populations and adaptive to northern/southern latitudes even one or more generations after the populations had been transplanted (to minimize non-genetic confounding effects; Kawecki and Ebert, 2004; Pazzaglia et al., 2021).
2. Genetic tests for outlier loci will identify genomic regions that have responded to natural selection in polar regions with exceptionally low allelic variation in 2 high-latitude populations and high genetic divergence from 2 low-latitude populations (Luikart et al., 2003; Narum and Hess, 2011). Functions associated with genes located in these regions are likely to have adapted genetically to polar conditions. For example, outlier tests identified genes related to growth and immunity as putatively adaptive to varying temperature and light conditions along latitudinal and bathymetric gradients in the seagrass *P. oceanica* (Jahnke et al., 2019).

In order to test whether the reaction norm of eelgrass is wide enough to acclimatize to a warming Arctic, we need to expose eelgrass to a simulation of near-future polar conditions. For example, seagrass meadows in Australia (*Halodule pinifolia* and *Halophila ovalis*) were shown to die at >38 days at 0.1mol photons m^−2^ d^−1^ (0.35% SI; Longstaff and Dennison, 1999). To determine the latitude at which eelgrass would lack sufficient daylight throughout the year, we would need an equivalent study that imitates future temperature and CO_2_ levels at different daylight lengths in a common-garden setup and, ideally, includes a variety of genotypes – since these can differ in their dark tolerance (Salo et al., 2015).

## Data Availability Statement

The datasets presented in this study can be found in online repositories. The names of the repository/repositories and accession number(s) can be found at:

https://figshare.com/projects/Adaptation_of_temperate_seagrass_to_Arctic_light_relies_on_seasonal_acclimatization_of_carbon_capture_and_metabolism/117825

https://www.ncbi.nlm.nih.gov/, PRJNA745558

https://dataview.ncbi.nlm.nih.gov/object/PRJNA745558?reviewer=d8qlenvsni8uvdohe3mik9n00s.

## Author Contributions

GH (project leader) and AJ planned the project and designed the sampling design. AJ, GH, and SA-H collected the shoots. AJ and BD analyzed the data and wrote the manuscript. JC performed the RNA extraction. MK and IS performed the library preparation and sequencing. SA-H, JO, and Z-MH were involved in data interpretation. All authors contributed to the article and approved the submitted version.

## Funding

This work was funded by the Norwegian Research Council (Havkyst project 243916). JC was supported by a visiting researcher grant from the Norwegian Research Council (project 281153). Research in the Marine and Environmental Sciences Centre (MARE) was funded by “Fundação para a Ciência e Tecnologia (FCT)” UIDB/04292/2020 and Mar2020 program (VALPRAD project, MAR-01.04.02-FEAMP-0007). BD was supported by FCT throughout an Investigator Contract (CEECIND/03869/2018).

## Conflict of Interest

The authors declare that the research was conducted in the absence of any commercial or financial relationships that could be construed as a potential conflict of interest.

## Publisher’s Note

All claims expressed in this article are solely those of the authors and do not necessarily represent those of their affiliated organizations, or those of the publisher, the editors and the reviewers. Any product that may be evaluated in this article, or claim that may be made by its manufacturer, is not guaranteed or endorsed by the publisher.

## Footnotes

^1^https://www.bioinformatics.babraham.ac.uk/projects/trim_galore/

^2^ http://www.bioinformatics.babraham.ac.uk/projects/fastqc/

^3^ https://github.com/gpertea/gffread
