# Supplementary material for: Adaptation of Temperate Seagrass to Arctic Light Relies on Seasonal Acclimatization of Carbon Capture and Metabolism
Source: Front Plant Sci. 2021 Dec 2;12:745855. doi: 10.3389/fpls.2021.745855 (PMC8675887; doi:10.3389/fpls.2021.745855)
Supplement: Supplementary file 1 [file Data_Sheet_1.docx]

Supplementary Material

# Supplementary Tables

**Supplementary Table 1**:

Environmental data collected at a high frequency over several months by data loggers at the Norwegian sampling site (Røvik) at two stations in the Thau Lagoon close to the French sampling site (Trombetta2019, Messiaen2020). The table lists the datalogger IDs, country, location, date and time of recordings, the measurement and its value. Measurments include temperature in °C, light intensity in Lux and PAR (µmol m^-2^ s^-1^), and Salinity in ‰.

Uploaded on figshare: [10.6084/m9.figshare.14955492](https://doi.org/10.6084/m9.figshare.14955492)

**Supplementary Table 2:** Measured and calculated parameters from the OJIP protocol of PAM measurements of each seagrass leaf. The following parameters are provided: Bckg: Background; F_0_: F_50μs_, fluorescence intensity at 50 μs; F_J_ = fluorescence intensity at J-step (at 2 ms); Fi: fluorescence intensity at i-step (at 60 ms); F_M_: maximal fluorescence intensity; F_V_: F_M_ - F_0_ (maximal variable fluorescence); V_J_: (F_J_ - F_0_) / (F_M_ - F_0_); V_i_:(F_i_ - F_0)_ / (F_M_ - F_0)_; F_M_ / F_0_; F_V_ / F_0_; F_v_ / F_M_; M_0_ or (dV/dt)_0_: TR_0_ / RC - ET_0_ / RC = 4 (F_300_ - F_0)_ / (F_M_ - F_0_); Area: Area between fluorescence curve and F_M_ (background subtracted); Fix Area: Area below the fluorescence curve between F_40μs_ and F_1s_ (background subtracted); S_M_: Area / (F_M_ - F_0_) (multiple turn-over); S_S_: the smallest S_M_ turn-over (single turn-over); N: S_M_ . M_0_ . (1 / V_J_) turn-over number Q_A_; Phi_P_0_: 1 – (F_0_ / F_M_) (or F_V_ / F_M_); Psi__0_: 1 – V_J_; Phi_E_0_: (1 – (F_0_ / F_M)_) . Psi__0_; Phi_D_0_: 1 – Phi_P_0_ – (F_0_ / F_M_); Phi_Pav = Phi_P_0_ (S_M_ / t_FM_) with t_FM_: time to reach F_M_ (in ms); ABS / RC: M_0_ . (1 / V_J_) . (1 / Phi_P_0_); TR_0_ / RC: M_0_ . (1 / V_J_); ET_0_ / RC: M_0_ . (1 / V_J_) . Psi__0_; DI_0_ / RC:(ABS / RC) – (TR_0_ / RC).

Uploaded on figshare: [10.6084/m9.figshare.14958810](https://doi.org/10.6084/m9.figshare.14958810)

**Supplementary Table 3**: Read counts. Read counts for 21,069 genes and 48 libraries. Gene IDs are listed in the first column, the mean coverage for each gene across all samples in the second column, and counts for all samples in the subsequent columns.

Uploaded on figshare: [10.6084/m9.figshare.14958837](https://doi.org/10.6084/m9.figshare.14958837)

**Supplementary Table 4**: Regularized log transformed read counts for 48 libraries at 13,932 genes with mean coverage >5. Gene IDs are listed in the first column, rlog values for all samples in the subsequent columns.

Uploaded on figshare: [10.6084/m9.figshare.14958867](https://doi.org/10.6084/m9.figshare.14958867)

**Supplementary Table 5**: Number of reads per library. Listed are the numbers of raw reads, trimmed reads, reads that mapped uniquely to nuclear DNA and organellar DNA of *Z*. *marina*, and reads that mapped to nuclear and organellar exons.

Uploaded on figshare: [10.6084/m9.figshare.14958936](https://doi.org/10.6084/m9.figshare.14958936)

**Supplementary Table 6**: Genes showing significant differences in gene expression between summer and winter. Columns specify the: 1. Population; 2. Upregulated.in, whether the gene was upregulated during summer or winter; 3. GeneID, the gene ID based on the *Zostera marina* genome annotation; 4. baseMean, the mean of normalized counts for all samples; 5. log2FoldChange, the log2 fold difference in normalized expression between samples taken at day and night (values > 1 indicate higher expression at day); 6. lfcSE, the standard error of the log2 fold difference; 7. stat, the Wald test statistic for differential expression; 8. pvalue, the p-value; 9. padj, the p-value adjusted by the Benjamini-Hochberg method to control for false discovery rate; 10. description, the gene description; 11. GO.labels, the associated Gene Ontology terms; 12. KO.Term, the associated KEGG Ontology terms.

Uploaded on figshare: [10.6084/m9.figshare.14958957](https://doi.org/10.6084/m9.figshare.14958957)

**Supplementary Table 7**: Genes showing significant differences in gene expression between day and night. Columns specify the: 1. Population; 2. Season, 3. Upregulated.in, whether the gene was upregulated during day or night; 4. GeneID, the gene ID based on the *Zostera marina* genome annotation; 5. baseMean, the mean of normalized counts for all samples; 6. log2FoldChange, the log2 fold difference in normalized expression between samples taken at day and night (values > 1 indicate higher expression at day); 7. lfcSE, the standard error of the log2 fold difference; 8. stat, the Wald test statistic for differential expression; 9. pvalue, the p-value; 10. padj, the p-value adjusted by the Benjamini-Hochberg method to control for false discovery rate; 11. description, the gene description; 12. GO.labels, the associated Gene Ontology terms; 13. KO.Term, the associated KEGG Ontology terms.

Uploaded on figshare: [10.6084/m9.figshare.14958990](https://doi.org/10.6084/m9.figshare.14958990)

**Supplementary Table 8**: KEGG metabolic pathways enriched with genes that were differentially expressed between day and night or between winter and summer in the French and/or Norwegian population of *Zostera marina*.

Uploaded on figshare: [10.6084/m9.figshare.14959008](https://doi.org/10.6084/m9.figshare.14959008)

# Supplementary Figures


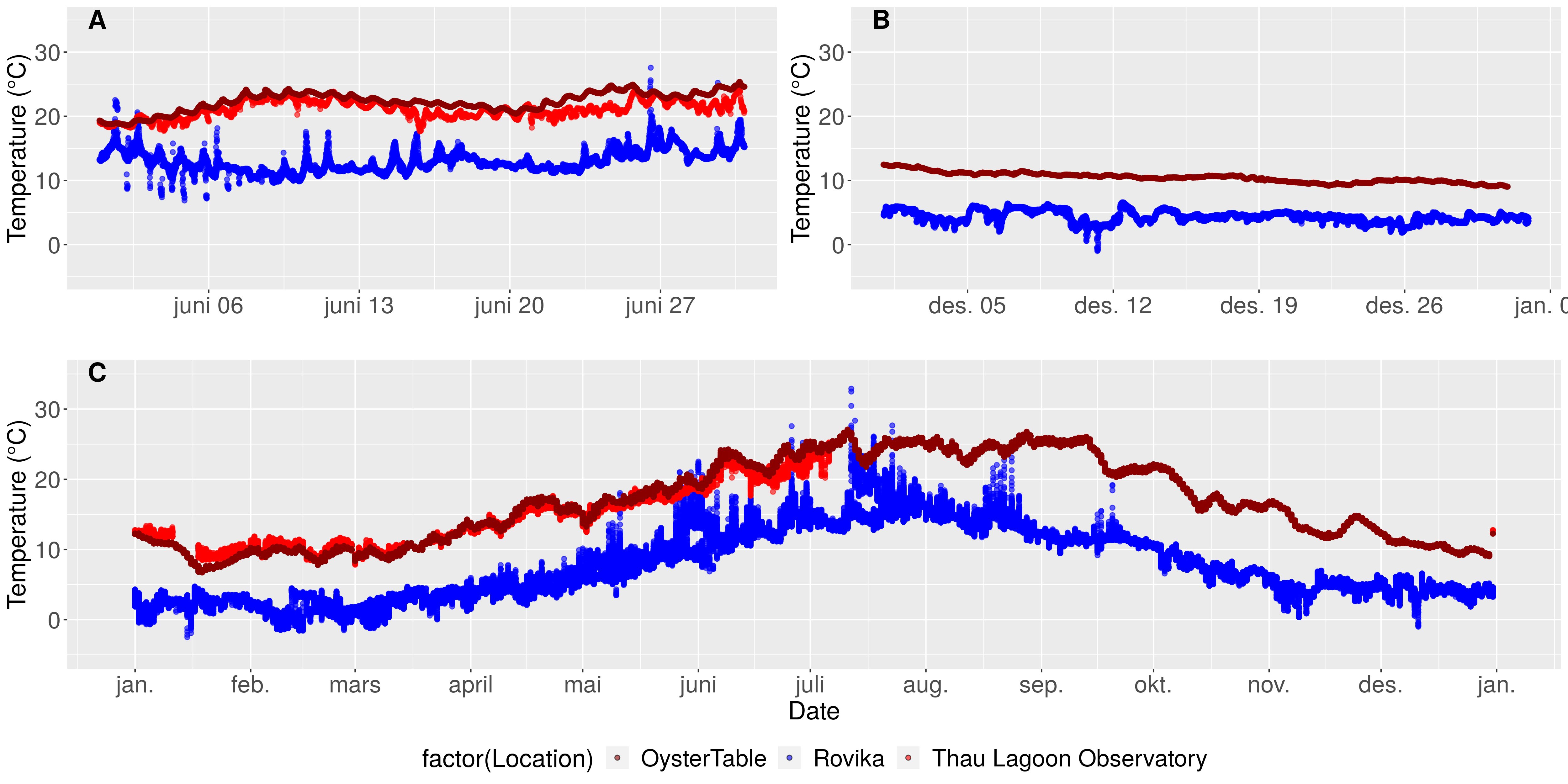
**Supplementary Figure 1.** Temperatures in June (a), December (b), and over the entire year (c) at the Norwegian sampling site (blue) and at two observatory stations in the Thau Lagoon close to the French sampling site (red, dark red).


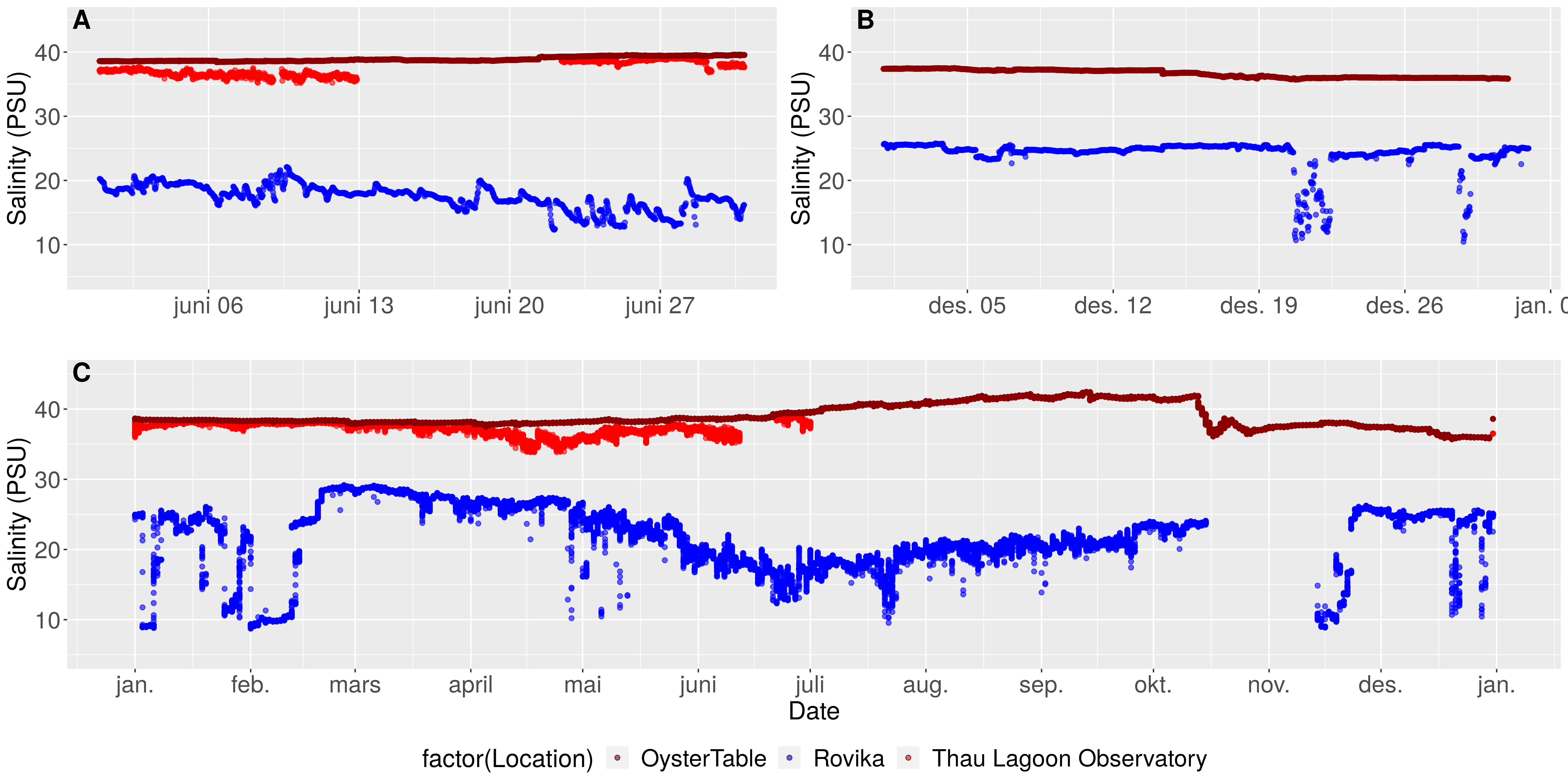
**Supplementary Figure 2.** Salinity levels in June (a), December (b), and over the entire year (c) at the Norwegian sampling site (blue) and at two observatory stations in the Thau Lagoon close to the French sampling site (red, dark red).


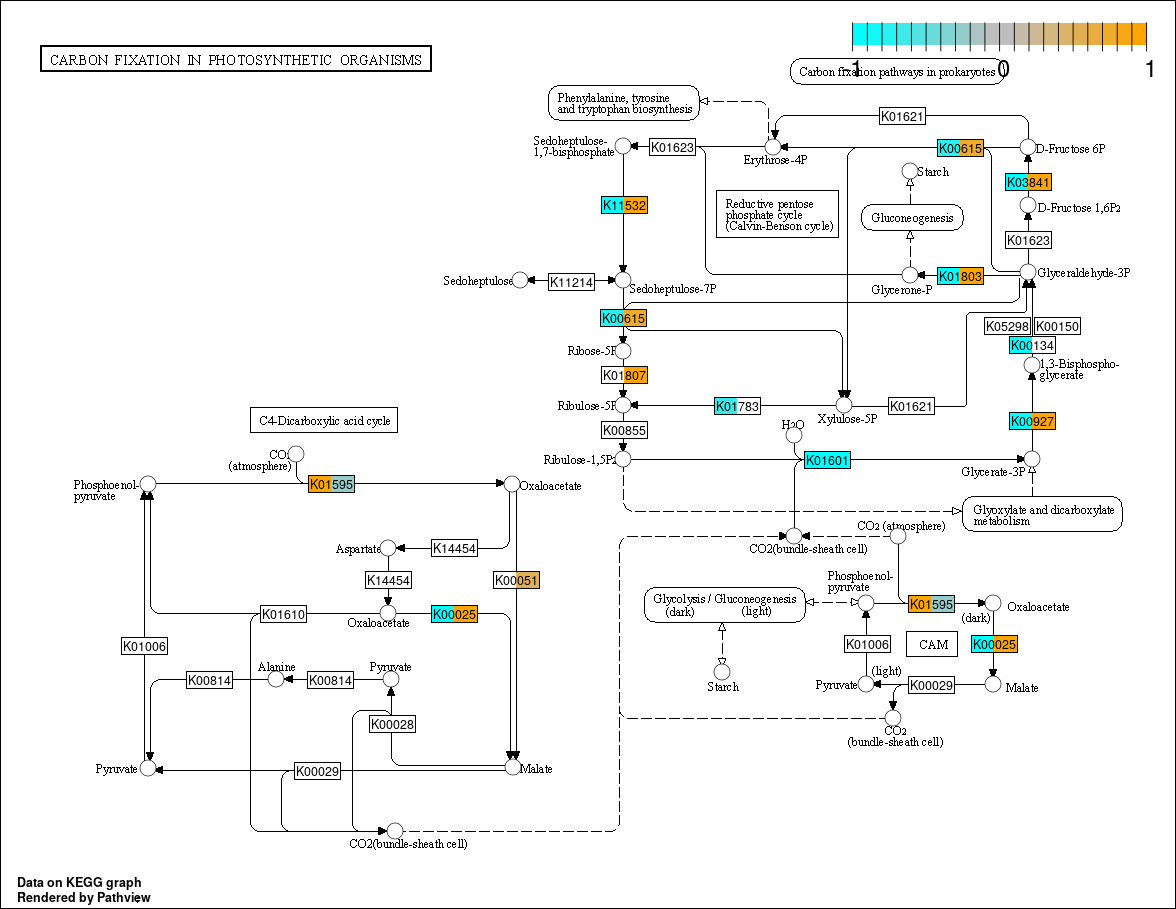


**Supplementary Figure 3.** Carbon fixation pathway showing seasonal expression changes (log2-fold, values <-1 or >1 are set to these limits) in gene functions (KEGG orthologies) that were significantly higher expressed in summer (orange) or winter (cyan) samples. Insignificant seasonal expression differences are shown in white. If several genes referred to the same KEGG orthology (KO) term, their average diurnal log2 fold change is shown. Each KO-term is partitioned into two sections corresponding to 1) French and 2) Norwegian samples.


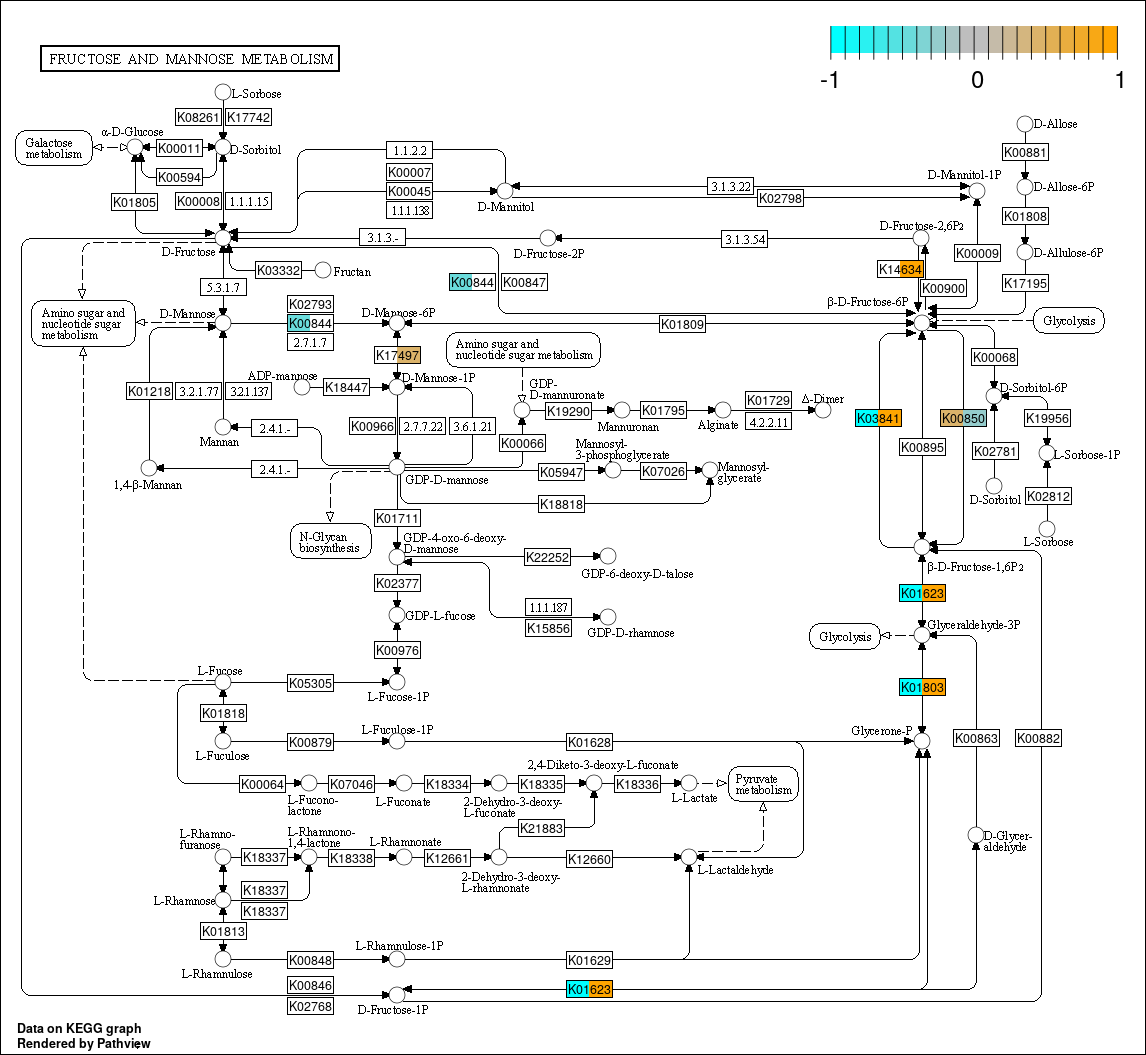


**Supplementary Figure 4.** Fructose and mannose metabolism pathway showing seasonal expression changes (log2-fold, values <-1 or >1 are set to these limits) in gene functions (KEGG orthologies) that were significantly higher expressed in summer (orange) or winter (cyan) samples. Insignificant seasonal expression differences are shown in white. If several genes referred to the same KEGG orthology (KO) term, their average diurnal log2 fold change is shown. Each KO-term is partitioned into two sections corresponding to 1) French and 2) Norwegian samples.


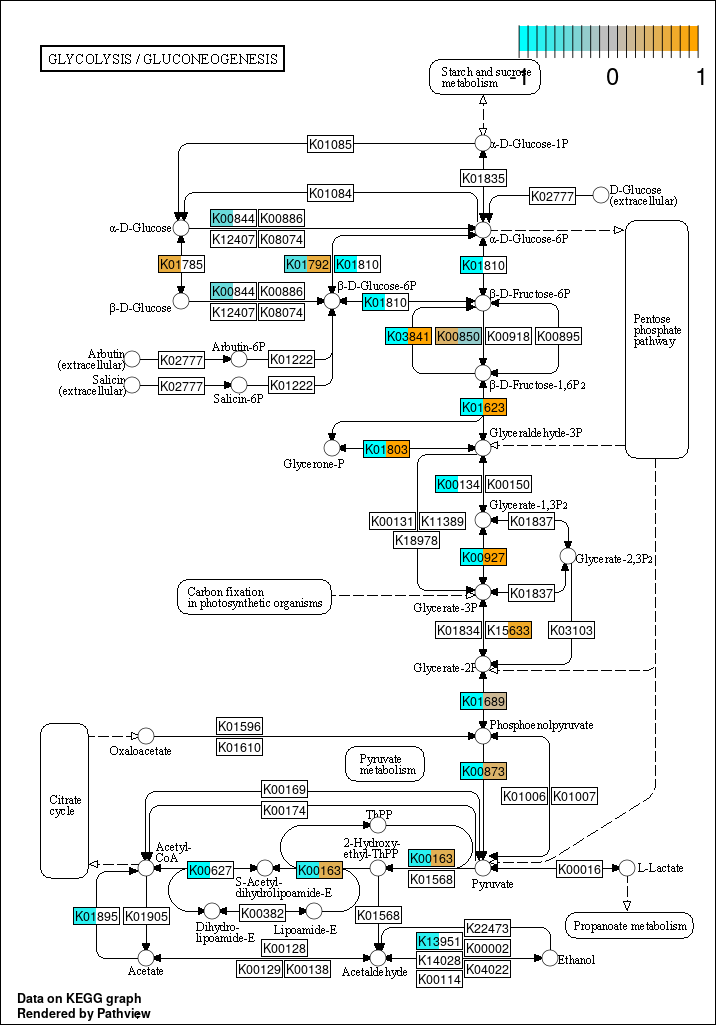
**Supplementary Figure 5.** Glycolisis/gluconeogenesis pathway showing seasonal expression changes (log2-fold, values <-1 or >1 are set to these limits) in gene functions (KEGG orthologies) that were significantly higher expressed in summer (orange) or winter (cyan) samples. Insignificant seasonal expression differences are shown in white. If several genes referred to the same KEGG orthology (KO) term, their average diurnal log2 fold change is shown. Each KO-term is partitioned into two sections corresponding to 1) French and 2) Norwegian samples.


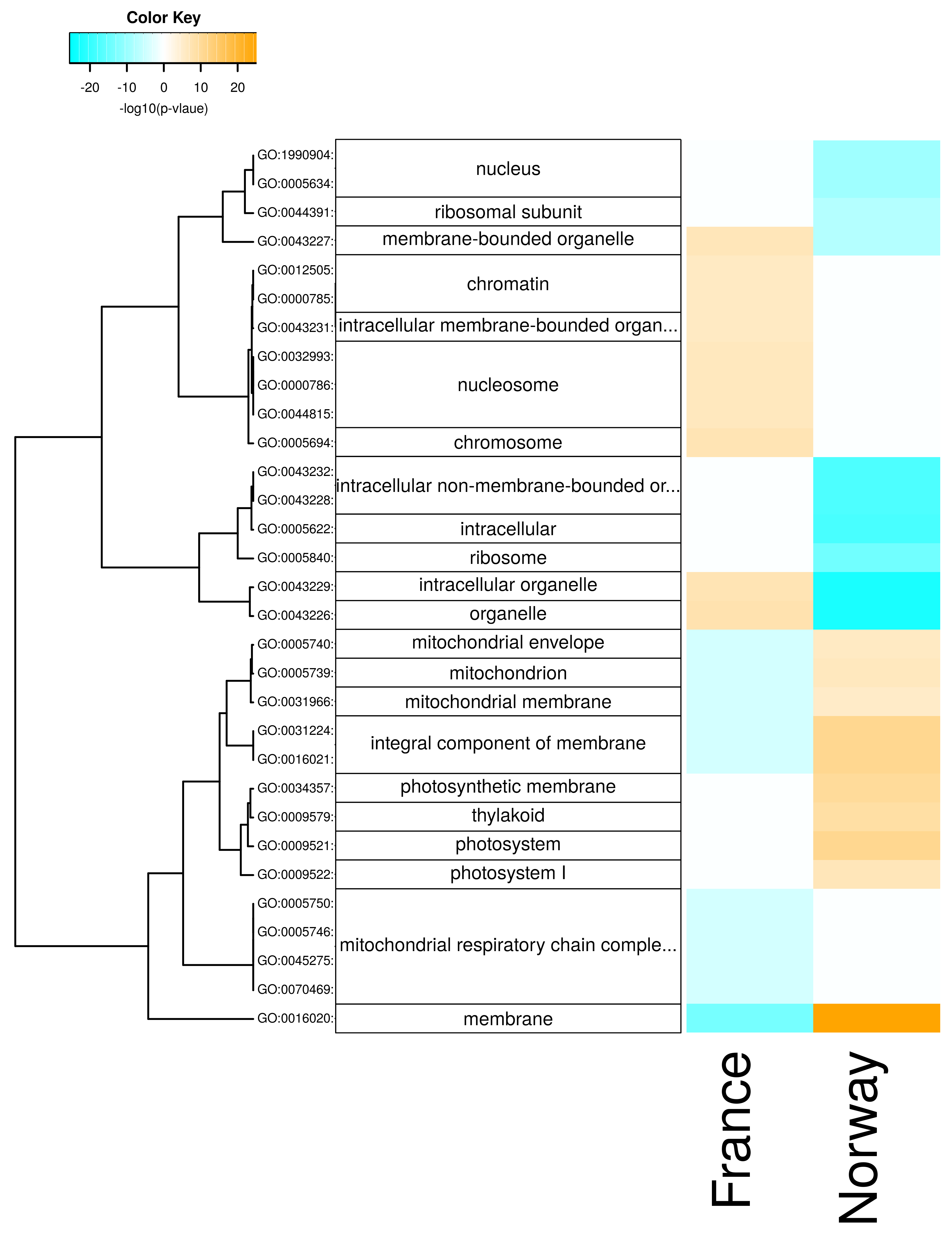


**Supplementary Figure 6.** Seasonal changes in cellular components. Enriched components are shown for each population with positive -log10(p-values) for genes upregulated in summer (orange) and with negative -log10(p-values) for genes upregulated in winter (cyan).


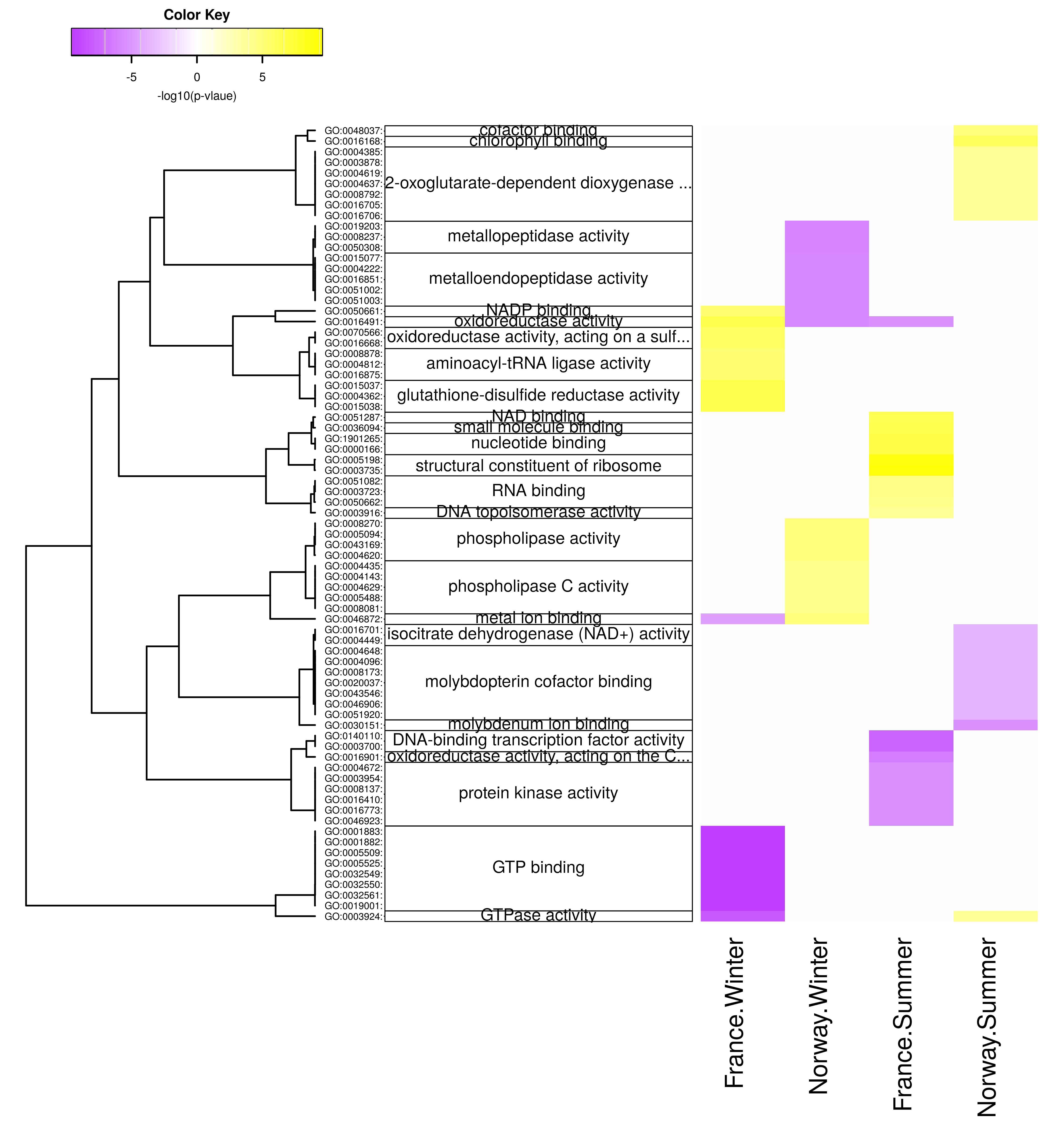


**Supplementary Figure 7.** Diurnal changes in molecular functions. Enriched functions are shown for each population x season with positive -log10(p-values) for genes upregulated at day (yellow) and with negative -log10(p-values) for genes upregulated at night (violet).


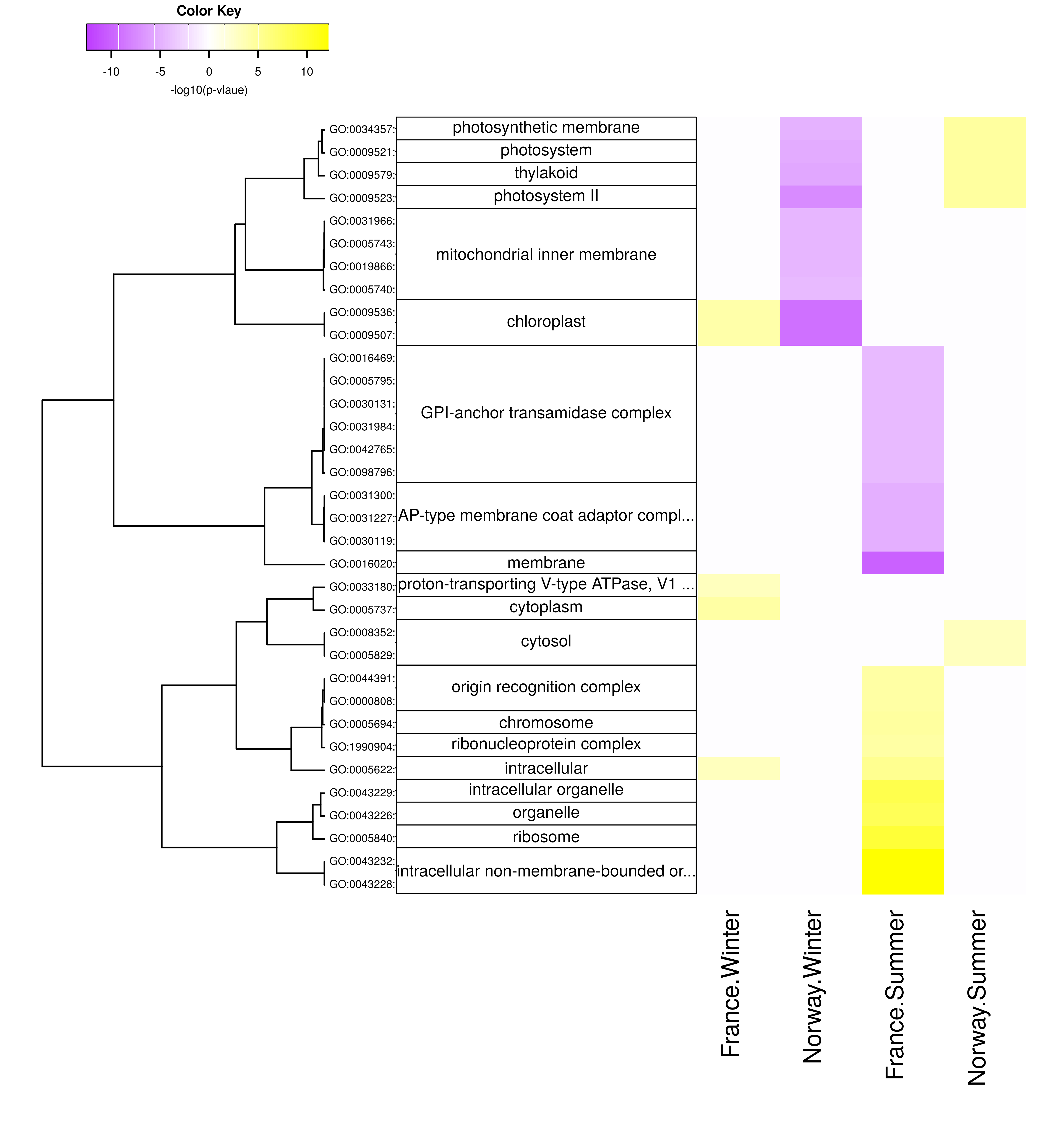


**Supplementary Figure 8.** Diurnal changes in cellular components. Enriched components are shown for each population x season with positive -log10(p-values) for genes upregulated at day (yellow) and with negative -log10(p-values) for genes upregulated at night (violet).


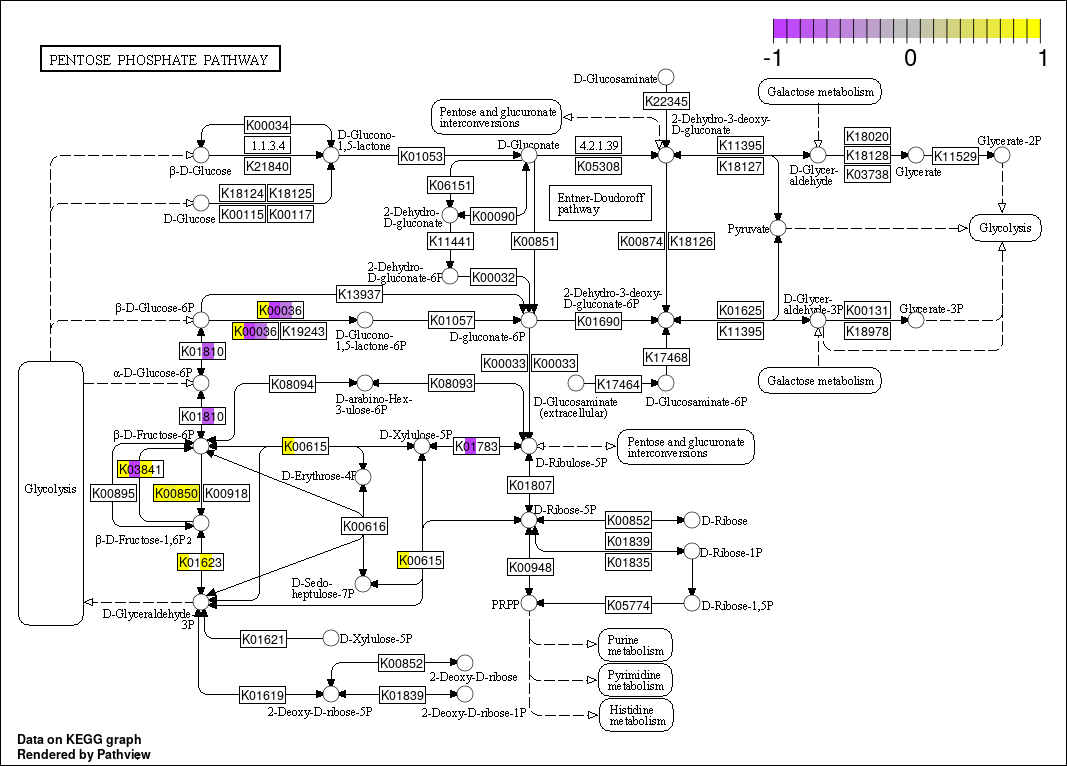


**Supplementary Figure 9.** Pentose phosphate pathway showing diurnal expression changes (log2-fold, values <-1 or >1 are set to these limits) in gene functions (KEGG orthologies) that were significantly higher expressed in day (yellow) or night (violet) samples. Insignificant expression differences between day and night are shown in white. If several genes referred to the same KEGG orthology (KO) term, their average diurnal log2 fold change is shown. Each KO-term is sliced into four pieces corresponding to 1) French winter samples, 2) Norwegian winter samples, 3) French summer samples, and 4) Norwegian summer samples.


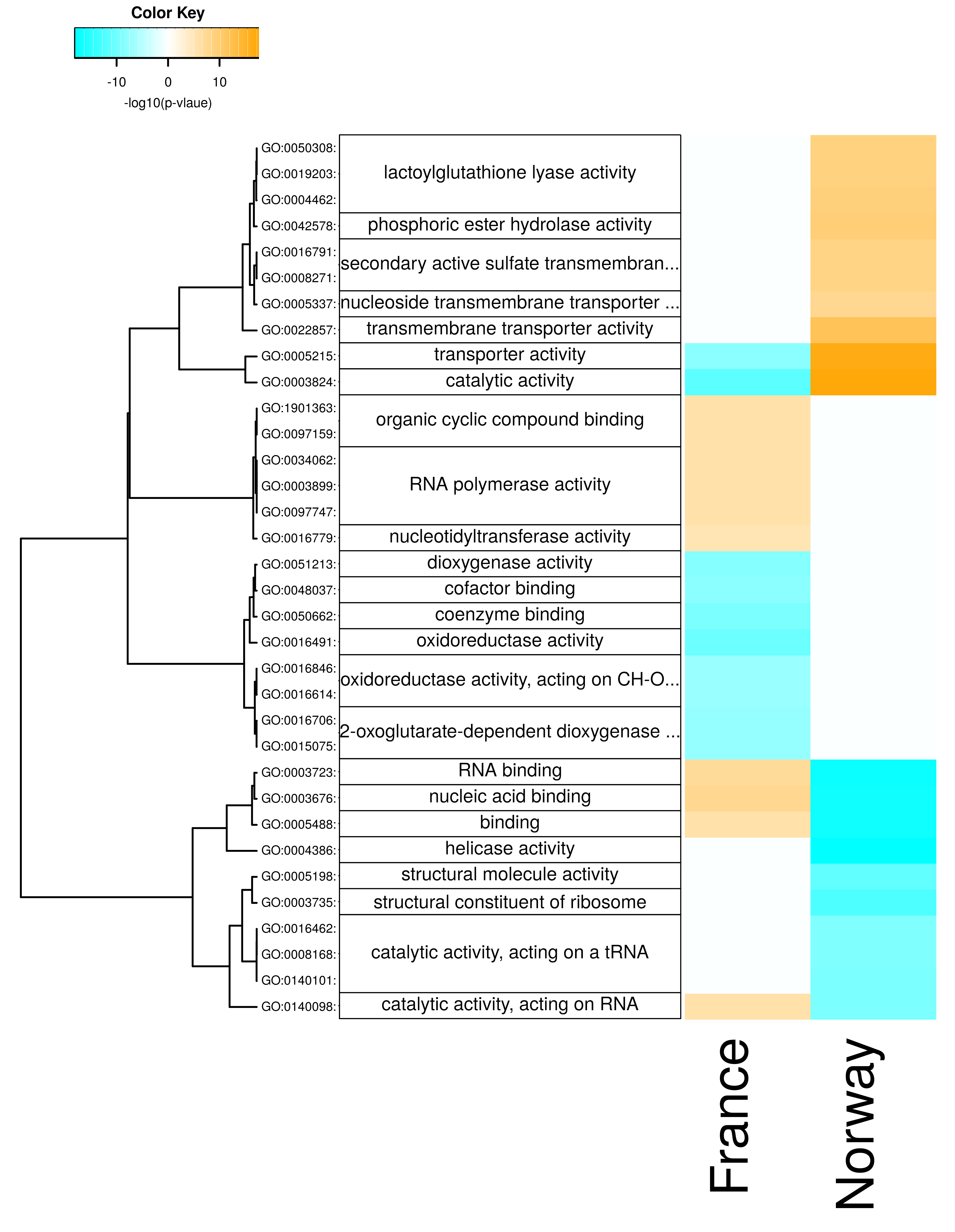


**Supplementary Figure 10.** Seasonal changes in molecular functions. Enriched functions are shown for each population with positive -log10(p-values) for genes upregulated in summer (orange) and with negative -log10(p-values) for genes upregulated in winter (cyan).


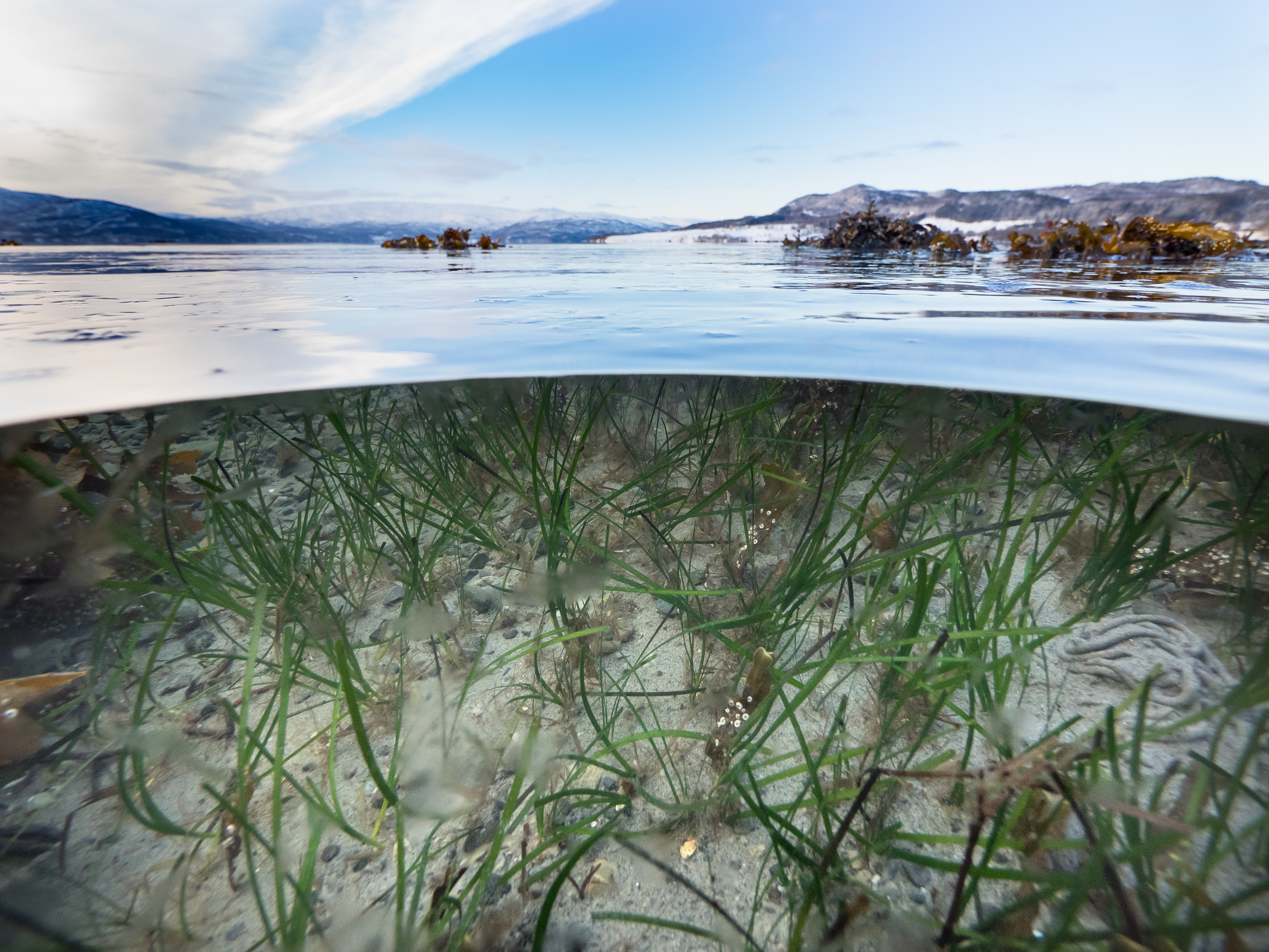


**Supplementary Figure 11.**
Eelgrass (Zostera marina) plants at our Norwegian sampling site (Røvik, 67°16’06.2" N, 15°15’38.4" E). The picture was taken on February 12^th^, 2016. The presence of fresh green leaves suggests that the plants had grown new shoots during the dark winter period.
